# Human transposons are an abundant supply of transcription factor binding sites and promoter activities in breast cancer cell lines

**DOI:** 10.1186/s13100-019-0158-3

**Published:** 2019-04-27

**Authors:** Jiayue-Clara Jiang, Kyle R. Upton

**Affiliations:** School of Chemistry and Molecular Biosciences, The University of Queensland, St Lucia, QLD 4072 Australia

**Keywords:** Transposable elements, Breast cancer, Transcription factor binding, Promoter, Epigenetics, Non-coding RNAs

## Abstract

**Background:**

Transposable elements (TE) are commonly regarded as “junk DNA” with no apparent regulatory roles in the human genome. However, a growing body of evidence demonstrates that some TEs exhibit regulatory activities in a range of biological pathways and diseases, with notable examples in bile metabolism and innate immunity. TEs are typically suppressed by epigenetic modifications in healthy somatic tissues, which prevents both undesirable effects of insertional mutagenesis, and also unwanted gene activation. Interestingly, TEs are widely reported to be dysregulated in epithelial cancers, and while much attention has been paid to their effects on genome instability, relatively little has been reported on their effects on gene regulation. Here, we investigated the contribution of TEs to the transcriptional regulation in breast cancer cell lines.

**Results:**

We found that a subset of TE subfamilies were enriched in oncogenic transcription factor binding sites and also harboured histone marks associated with active transcription, raising the possibility of these subfamilies playing a broad role in breast cancer transcriptional regulation. To directly assess promoter activity in triple negative breast cancer cell lines, we identified four breast cancer-associated genes with putative TE-derived promoters. TE deletion confirmed a contribution to promoter activity in all cases, and for two examples the promoter activity was almost completely contained within the TE.

**Conclusions:**

Our findings demonstrate that TEs provide abundant oncogenic transcription factor binding sites in breast cancer and that individual TEs contain substantial promoter activity. Our findings provide further evidence for transcriptional regulation of human genes through TE exaptation by demonstrating the regulatory potential of TEs in multiple breast cancer cell lines.

**Electronic supplementary material:**

The online version of this article (10.1186/s13100-019-0158-3) contains supplementary material, which is available to authorized users.

## Background

Transposable elements (TE), or transposons, are repetitive genetic elements that are ubiquitous in eukaryotic genomes [1, 2]. When first discovered in maize by Barbara McClintock in the mid-1940s, TEs were proposed to be “controlling elements” capable of regulating gene activity [3, 4]. McClintock’s theory was initially dismissed, and the prevailing view was that TEs were “junk” or “selfish” DNA sequences with no apparent regulatory roles [5–7]. However, in more recent years, McClintock’s theory of TEs as gene expression regulators has been revised and refined by emerging evidence showing that they do indeed play a role in modulating and reshaping host transcriptional regulatory networks [8–11]. In fact, the regulatory roles of TEs are not rare events exclusive to plants, but are common to almost all eukaryotic evolutionary lineages, including humans [12–14].

The regulatory activity of TEs is derived from the cis-regulatory elements within their sequences, which include internal promoters and binding sites that can be recognised by host transcription factors (TF) and RNA polymerases (RNA Pol) [2, 8, 10, 15–17]. For example, LTR retrotransposons, which originated from retroviral infections that successfully integrated into the host germline, originally contained an RNA Pol II promoter in each of their long terminal repeats (LTR) [2]. This compatibility is essential for the transcription of TEs, and allows them to exploit host machinery to aid their proliferation in the host genome [2]. While TEs directly benefit from host compatibility, the host also stands to benefit through exaptation of TE sequences to modify or create transcriptional networks for the regulation of host genes. During the course of evolution, many TEs remaining in the human genome have been exapted to contribute promoters or enhancers of human genes [9, 15, 18, 19]. In this study, we focus specifically on the promoter activity of TEs in the human genome.

Human TEs harbour a substantial number of TF binding sites (TFBS). On average, TEs are estimated to contain ~ 19% of the total TFBSs within the human genome [8]. These binding sites are recognised by a diverse group of TFs, differing greatly in their biological functions. Amongst these are components of the basic transcription machinery, such as the TATA box binding protein (TBP), as well as factors involved in the remodelling of chromatin states, such as the chromodomain helicase DNA-binding protein 1 (CHD1) and CHD2 [8]. In addition, some pathway-specific TFs have also been demonstrated to rely on TEs for their binding sites. A recent example is the interferon regulatory factor (IRF), and signal transducer and activator of transcription (STAT) TFs involved in the innate immunity [10].

Furthermore, an early study by Jordan et al. shows that 25% of the > 2000 human promoters documented in The Human Promoter Database contain TE-derived DNA sequences [15]. This finding is supported by recent advances in the Cap Analysis of Gene Expression (CAGE) technology, which maps genome-wide transcription start sites (TSS) by identifying mRNA 5′ end clusters [20]. A defining feature of promoters is the presence of transcription initiation sites, and CAGE data demonstrate that approximately 24% of TSSs (defined by the presence of two or more CAGE tags) are located within TEs [18]. Several studies have identified individual TEs with promoter functions in the genome. One example is MER11A, an ancient LTR acting as the primary promoter to *BAAT*, which encodes an enzyme involved in bile metabolism [21, 22]. Further examples are antisense L1 and Alu elements that together contribute the sole apparent promoters to *HYAL-4* and *FUT5*, which are involved in hyaluronan catabolism and cell adhesion respectively [9]. These examples demonstrate the presence of TE-derived promoter activity in a diverse range of biological pathways.

While these cis-regulatory sequences have endowed TEs with the potential to regulate human gene expression, the transcriptional activity of the majority of TEs are suppressed heavily in somatic tissues [23, 24]. This is hypothesised to be a defence mechanism by the host genome, which protects the host from large mutations resulting from active TE mobilisation, as well as abnormal gene expression driven by TE-derived promoters and enhancers [23–25]. The human genome employs an array of mechanisms to defend against TE activation, and the most relevant to transcriptional repression are DNA methylation and histone tail modifications [25–27].

Increased DNA methylation is often associated with gene silencing, and most TEs are heavily suppressed by DNA methylation and thus rendered inactive in somatic tissues [27–29]. Unlike DNA methylation, the relationship between human TEs and histone modifications is highly complex and less well-understood. Kondo and Issa first showed the recruitment of repressive H3K9 methylation to Alu elements in the human genome [26]. However, a study by Huda et al. on CD4+ T cells shows that Alu and L2 elements are enriched in active marks, while L1 and LTR TEs are depleted for active marks, and/or enriched for repressive marks [25]. On the other hand, TEs which are exapted to perform regulatory functions are often associated with active histone marks, resembling those typically associated with active regulatory regions [10, 30].

However, the suppressive regulations over TEs, such as DNA methylation, are often alleviated in some disease states, and the transcriptional activation of TEs is commonly observed in tumours [28, 31, 32]. The oncogenic capacity of TE-derived transcriptional regulation is summarised with specific examples in recent reviews [32, 33]. For example, TE-derived promoters have been documented to lead to oncogene activation, followed by neoplastic transformation and disease progression in some types of cancer, such as Hodgkin’s lymphoma, bladder tumours and diffuse large B cell lymphoma [34–36]. However, the involvement of TE-derived promoters in other cancer types remains largely unknown. In this study, we aimed to address this gap in knowledge by investigating the activity of TE-derived promoters in breast cancer cell lines. More specifically, we focussed on the highly malignant triple negative breast cancer (TNBC) subtype, which lacks the expression or amplification of hormone receptors, and is thus refractory to targeted therapies [37–40].

In this study, we aimed to investigate the transcriptional activity of TEs in breast cancer, particularly their interactions between TFs and contribution to promoter activity, using breast cancer cell lines as a model. We analysed genome-wide binding sites for three breast cancer-associated TFs (C/EBPβ, E2F1 and MYC) in MCF7 cells, and confirmed that a substantial fraction of these TFBSs resided in TEs. We showed that these TFBSs were distributed across a diverse range of TEs, and identified enriched TE subfamilies with a potentially important role in modulating breast cancer transcription. We found that the binding of breast cancer-associated TFs to TEs was correlated with active histone modifications, further supporting the transcriptional activity of these TEs. We subsequently identified individual TEs within the promoters of breast cancer-associated genes and confirmed that TEs located upstream of *SYT1, UCA1, AK4* and *PSAT1* contributed promoter activity in TNBC cell lines.

## Results

### TEs were an abundant source of breast cancer-associated TFBSs

To investigate the prevalence of TEs in breast cancer-associated TFBSs, we mapped the genome-wide binding sites for C/EBPβ, E2F1 and MYC in MCF7 breast cancer cells by re-analysing publicly available chromatin immunoprecipitation sequencing (ChIP-seq) datasets. These TFs were selected for analysis based on literature evidence on their oncogenic capacity in cancer [37, 41–47]. In each TF dataset, high confidence ChIP-seq peaks were called using HOMER, with a false discovery rate threshold of 0.001 [48]. We mapped 26,934 to 101,382 binding sites for each TF, with up to ~ 55% of these TFBSs identified to overlap TEs (Table 1). In total, 93,901 TE genomic locations, containing TEs from 1069 subfamilies, were found to harbour at least one breast cancer-associated TFBS.

**Table 1:** Summary of ChIP-seq data analyses of breast cancer-associated TFBSs [64, 81]

TF	Data source	Total peak number	Peaks in TEs (% of total)
C/EBPβ	ENCODE	54,182	29,579 (54.6%)
E2F1	GEO	26,934	9748 (36.2%)
MYC	GEO	101,382	35,704 (35.2%)
MYC	ENCODE	61,454	15,936 (25.9%)

**Fig. 1:**
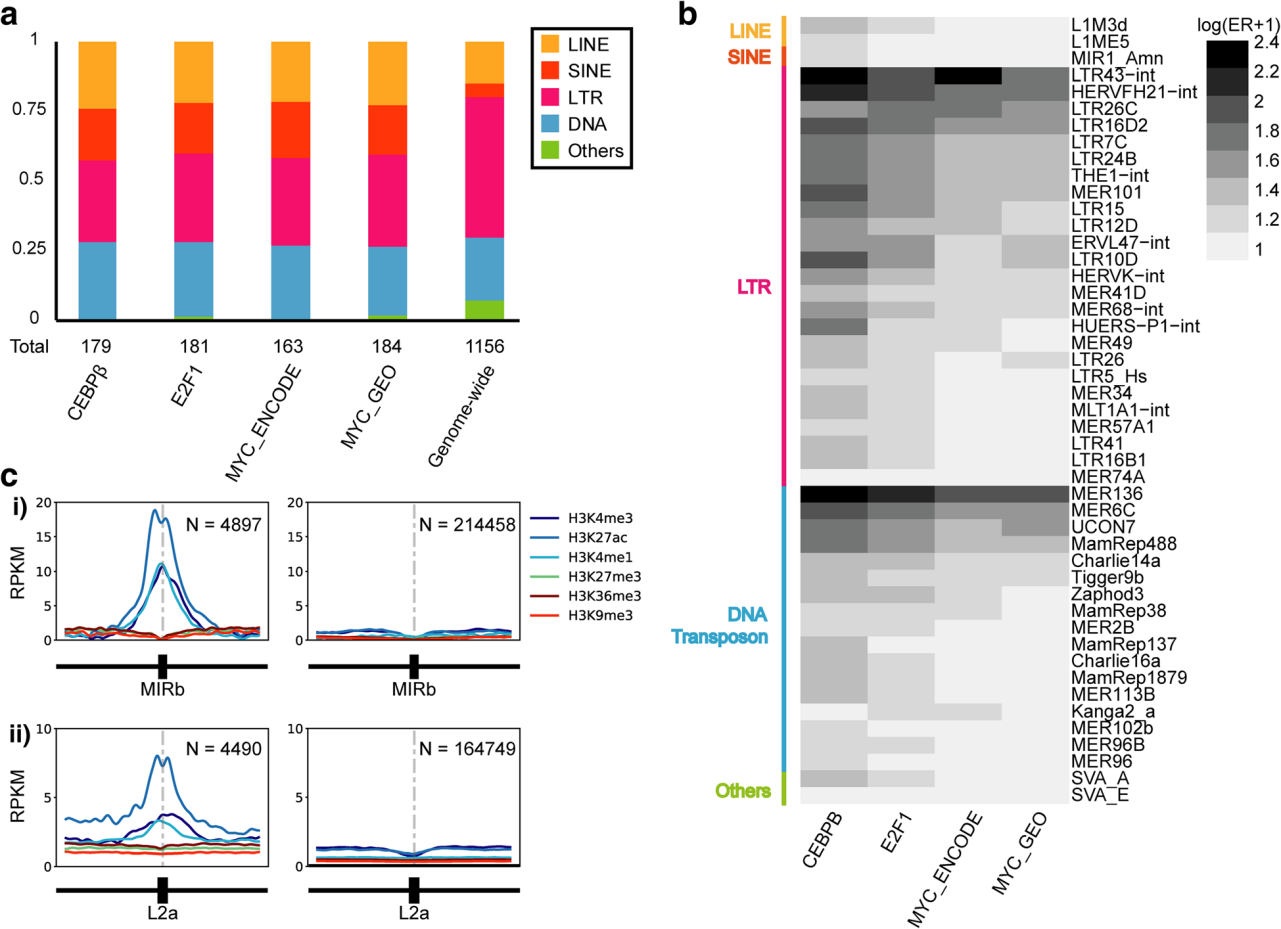
Enriched TE subfamilies in breast cancer-associated TFBSs. **a** 268 out of 1156 TE subfamilies, with representatives from all major classes, were found to be significantly enriched in breast cancer-associated TFBSs, as identified by a binomial test, where *p* < 4.3E-5 indicated statistical significance. The abundance of TE subfamilies in the human genome, categorised into classes, are shown for reference. **b** Heatmap demonstrates the level of enrichment of the most highly enriched TE subfamilies (> 7.5 enrichment ratio in all TF datasets) (ER = Enrichment Ratio). **c** Subfamilies enriched in the binding sites of C/EBPβ, E2F1 and MYC were investigated for their association with histone marks (H3K27ac, H3K4me1, H3K4me3, H3K36me3, H3K9me3 and H3K27me3) in MCF7 cells using published ChIP-seq datasets. The left panels display the histone modification profile of TE elements containing TFBSs, while the right panels display the profile of TE elements lacking TFBSs. The average RPKM values at 50 bp resolution over a 10 kb region centred on the TEs (filled ractangles) are plotted for i) MIRb and ii) L2a

We then investigated whether individual TE subfamilies were enriched for breast cancer-associated TFBSs compared to their expected coincidence. Binomial tests revealed that 268 individual TE subfamilies, with representatives from all major classes, were found to be significantly enriched in at least one ChIP-seq dataset (*p* < 4.3E-5 indicated statistical significance). LTRs were observed to contribute more enriched subfamilies than any other class of TEs for all TF datasets investigated, and represented approximately 30% of enriched TE subfamilies (Fig. 1a). DNA transposons contributed approximately 26% of the enriched subfamilies (Fig. 1a), second only to LTRs. The extent of enrichment for highly enriched subfamilies are shown in Fig. 1b as representative results, and all enriched subfamilies are shown in Additional file 1: Figure S1-S5.

### TF binding in TEs was correlated with active epigenetic signatures

We next sought to determine whether these TE subfamilies were likely to be transcriptionally active in breast cancer by re-analysing publicly available ChIP-seq datasets for active (H3K27ac, H3K4me1, H3K4me3 and H3K36me3) and repressive (H3K9me3 and H3K27me3) histone tail modifications in MCF7 cells [49–53]. For each of four highly enriched TE subfamilies (MIRb, L2a, AluJb and L2b), active epigenetic signatures were consistently observed for TE elements bound by TFs, but were not observed for elements of the same subfamily that lacked binding (Fig. 1c and Additional file 1: Figure S6). Repressive signatures were not observed in either bound or unbound subgroups for any TE subfamily (Fig. 1c and Additional file 1: Figure S6).

### A subset of breast cancer-associated mRNAs and lncRNAs were identified to have putative TE-derived promoters

**Fig. 2:**
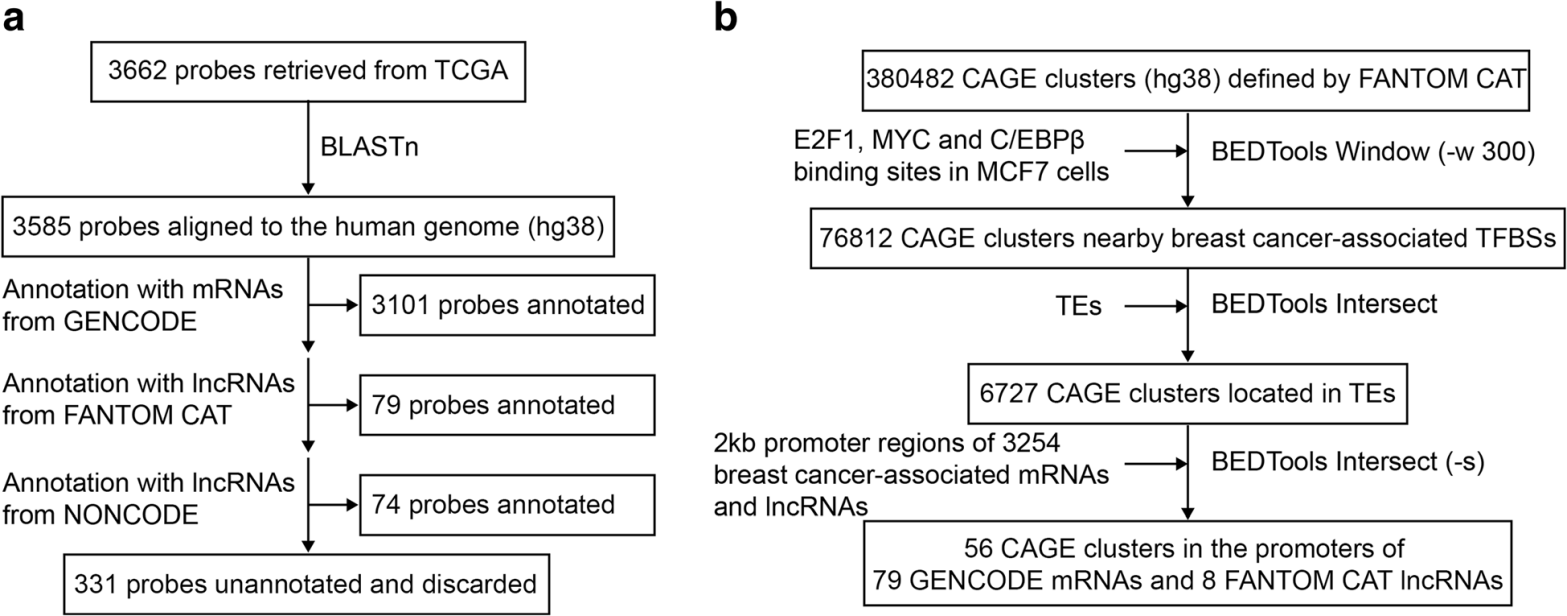
Identification of breast cancer-associated mRNAs and lncRNAs using published microarray data. **a** Microarray probes identified by TCGA as identifying differentially expressed genes in breast cancer were re-annotated with human mRNAs and lncRNAs using a hierarchical process [56–58]. 3585 out of 3662 probes were aligned to the human genome (hg38) by BLASTn [55]. The targets of the aligned probes were annotated by intersecting probe locations with GENCODE mRNAs, FANTOM CAT lncRNAs and NONCODE lncRNAs in order of descending priority [56–58]. 3101 probes were annotated with mRNAs [58]. 79 probes were annotated with lncRNAs from FANTOM CAT [56]. 74 probes were annotated with lncRNAs from NONCODE [57]. Probes that could not be aligned or annotated were discarded from downstream analysis. **b** Breast cancer-associated genes with putative TE-derived promoters were identified by a hierarchical process. TSSs, indicated by CAGE clusters defined by FANTOM CAT [56] were intersected with breast cancer-associated TFBSs using a 300 bp window, followed by intersecting with TEs and promoters of breast cancer-associated mRNAs and lncRNAs. 41 genes (79 mRNAs) and 8 FANTOM CAT lncRNAs were identified to have putative TE-derived promoters

Next, we sought to identify breast cancer-associated genes with putative TE-derived promoters. The Cancer Genome Atlas Network (TCGA) had previously identified 3662 genetic elements as being differentially expressed in breast cancer [54, 55]. From the microarray probes for these elements, 3585 could be aligned to the human genome (hg38) with BLASTn [54, 55]. The probe targets were annotated with GENCODE mRNAs, as well as FANTOM CAT and NONCODE long non-coding RNAs (lncRNA) using a hierarchical process [56–58] (Fig. 2a). In total, 3101 probes were annotated with mRNAs from the GENCODE database [58] (Fig. 2a). An additional 79 and 74 probes were annotated with lncRNAs from the FANTOM CAT and NONCODE databases respectively [56, 57] (Fig. 2a).

We then identified genes and lncRNAs with putative TE-derived promoters by intersection with TSSs and TFBSs. Of the ~ 380,000 FANTOM CAT CAGE clusters, ~ 76,800 clusters were located nearby a breast cancer-associated TFBS (window = 300 bp). Amongst these CAGE clusters, 6727 overlapped with TEs. Finally, 56 TE-harboured CAGE clusters were located within the putative promoters for 41 breast cancer-associated genes (identified by 79 GENCODE mRNA transcripts) and 8 FANTOM CAT lncRNAs (for list of CAGE clusters and genes see Additional file 2). TEs with putative promoter activity are listed in Additional file 3.

A literature search was performed for genes and lncRNAs with putative TE-derived promoters to identify those with existing experimental evidence for oncogenic activity. *SYT1, UCA1, AK4* and *PSAT1* were selected for downstream analysis (Table 2) (literature evidence summarised in Additional file 4: Table S1). In particular, *UCA1* encodes an oncogenic lncRNA shown to disrupt multiple tumour suppressive mechanisms. For example, it is found to inhibit the tumour suppressive miR-143 by direct binding, and inhibit translation of the p27 tumour suppressor by competitive inhibition [59, 60]. The tumour-specific expression of *UCA1* is a potential biomarker for bladder and pancreatic cancer [61–63].

**Table 2:** Summary of candidate genes, TEs and TFs

Gene name	TE class	TE family	TE subfamily	TF
*SYT1* (Synaptotagmin I)	LINE	L1	L1PA2	E2F1
*UCA1* (Urothelial carcinoma associated 1)	LTR	ERV1	LTR7C	C/EBPβ
*AK4* (Adenylate kinase-4)	SINE	MIR	MIRb	E2F1
*PSAT1* (Phosphoserine aminotransferase 1)	SINE	MIR	MIR3	E2F1

### TEs contributed significant promoter activity to breast cancer-associated genes

For *SYT1, UCA1, AK4* and *PSAT1,* the promoter activity was estimated via luciferase assays in three TNBC cell lines (MDA-MB-468, MDA-MB-231 and BT549). To estimate the contribution of TEs to the promoter activity, TEs hypothesised to harbour promoter activity were removed from the wild-type promoters, and the remaining DNA sequences within the promoters were tested for promoter activity by luciferase assays. TE deletion resulted in reduced activity of all four promoters in at least one cell line.

**Fig. 3:**
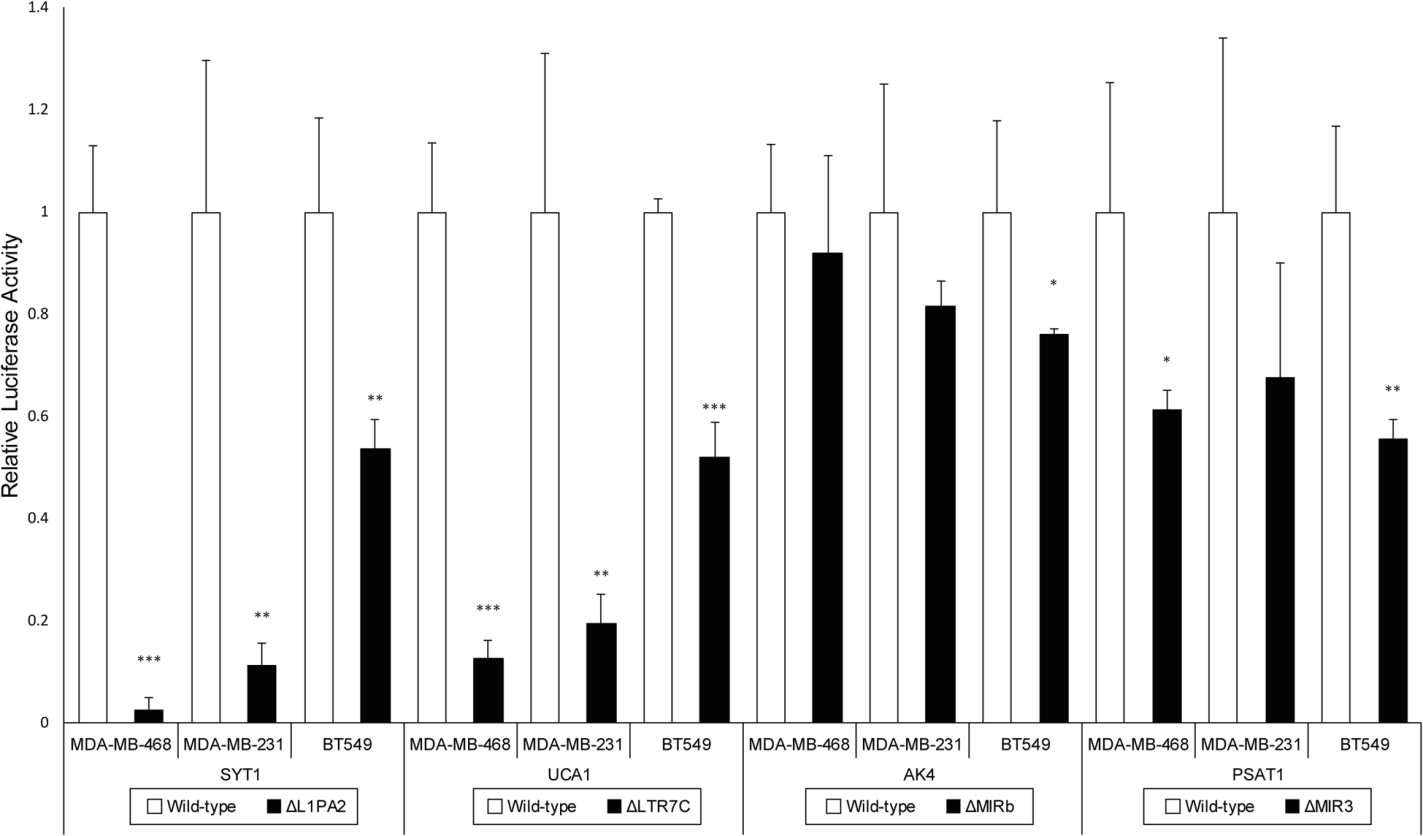
The effect of TE deletion on relative luciferase activity in MDA-MB-468, MDA-MB-231 and BT549 cells. Luciferase assays were performed for *SYT1*, *UCA1*, *AK4* and *PSAT1* in 3 independent experiments (*n* = 3) with triplicates. Bar graphs represent the mean relative luciferase activities, with white bars for wild-type and black bars for TE deletion. Error bars represent the standard deviations among independent experiments. The relative luciferase activity of TE-deleted constructs was compared against the corresponding wild-type using a one-tailed t-test, assuming equal variances. *p < 0.05, **p < 0.01, ***p < 0.001

Strikingly, deletion of L1PA2-*SYT1* resulted in near complete ablation of expression in MDA-MB-468 and MDA-MB-231 cells, where only 2.6 and 11.2% of promoter activity remained after deletion respectively (Fig. 3). A significant decrease in promoter activity was also observed in BT549 cells, with 53.7% of activity remaining after L1PA2-*SYT1* deletion. Similar results were observed for the deletion of LTR7C*-UCA1*. Relative luciferase activity was reduced to 12.6 and 19.4% in MDA-MB-468 and MDA-MB-231 cells respectively, and a 52% remaining promoter activity was observed in BT549 cells (Fig. 3). For *AK4*, TE deletion resulted in a trend of reduced promoter activity; however, statistical significance was only achieved in BT549 cells (Fig. 3). In addition, the promoter activity of *PSAT1* showed a significant decrease in BT549 and MDA-MB-468 cells following the removal of TE, where only 55.5–61.2% of promoter activity remained (Fig. 3). Although less than 70% of promoter activity was observed in MDA-MB-231 cells following MIR3*-PSAT1* removal, the significance of the effect was likely masked by the variability present (Fig. 3).

### TE-derived promoter activity was correlated with epigenetic derepression

To further characterise these TE-derived promoters, we investigated whether they displayed differential methylation in breast cancer tumours and whether they resided in open chromatin in breast cancer cell lines, by analysing published whole-genome methylation capture sequencing and DNase-seq datasets [64, 65] (for DNase-seq data source see Additional file 4: Table S2). L1PA2-*SYT1* was found to be hypomethylated in TNBC tumours (t_5_ = − 2.71, *p* = 0.021). Reduced methylation in tumours was not observed for the other TEs of interest. Investigating published DNase-seq data as an indicator of open chromatin, we observed significantly higher DNase sensitivity in L1PA2-*SYT1* and LTR7C*-UCA1* elements in MCF7 cells, compared to HMEC control cells (χ^2^_1_ = 41.97, *p* = 4.64 × 10^− 11^ and χ^2^_1_ = 7.88, *p* = 0.0025 respectively), while the remaining TEs showed no statistically significant increase in DNAse sensitivity in MCF7 cells.

## Discussion

TEs have been demonstrated to be an abundant reservoir of cis-regulatory sequences compatible with human transcription machinery [2, 8–10, 18, 22]. They supply a substantial number of TFBSs and promoters to human genes, and thereby modulate gene expression in normal biological pathways as well as disease progression [8, 9, 32]. While most TEs are under tight, suppressive regulation in somatic tissues, they often escape epigenetic repression in cancer and contribute promoters which cause abnormal oncogene expression [25–28, 31, 32]. TE-derived promoters have been found to drive tumorigenic gene expression in Hodgkin’s lymphoma, bladder tumours and diffuse large B cell lymphoma [34–36]; however, the contribution of TE-derived promoters to breast cancer transcriptional regulation is poorly studied. Here, we addressed this gap in knowledge by investigating the landscape of TF binding of TEs, as well as identifying and validating oncogene-associated TE-derived promoters in breast cancer cell lines.

Human TEs have been reported to contain binding sites for TFs, with activities ranging from general transcription initiation (e.g. TBP) to chromatin remodelling (e.g. CHD1 and CHD2), as well as those with specialised roles in cellular functions (e.g. IRF and STAT involved in innate immunity) [8, 10]. Here, we investigated the extent to which TEs contributed binding sites for three breast cancer-associated TFs (C/EBPβ, E2F1 and MYC) in breast cancer. On average, TEs contributed ~ 38% of the binding sites in MCF7 cells, and up to 54.6% of binding sites for C/EBPβ (Table 1). This demonstrates that TEs represent an abundant source of breast cancer-associated TFBSs.

TEs can be categorised hierarchically into classes, families and subfamilies based on their sequence features [1]. Although many TEs originally contained cis-regulatory sequences that were compatible with the host transcriptional machinery, their sequence can be truncated during initial insertion, and acquired mutations can degrade these cis-regulatory sequences [2]. In particular, LTR retrotransposons are an abundant source of cis-regulatory sequences and are often exapted for the regulation of human genes [8, 10]. The pervasive regulatory activity of LTRs can be explained by the fact that 85% of the LTR retrotransposons in the human genome consist of long terminal repeats only, which contain the original RNA Pol II promoter sequences [1, 2]. On the contrary, the majority of human DNA transposons exist in the form of miniature inverted-repeat transposable elements (MITE), many of which lack cis-regulatory elements such as internal promoters [2, 66]. Of the total 1156 TE subfamilies investigated, we identified 268 subfamilies with significant enrichment for breast cancer-associated TF binding. Notably, LTRs represent 30% of all significantly enriched subfamilies (Fig. 1a). While we observe enrichment for TF binding within some LTR subfamilies, we do not observe a general enrichment of LTRs as a class of TEs (50.3% of all TE subfamilies). Surprisingly, DNA transposons contributed ~ 26% of the enriched TE subfamilies for all TFs investigated (compared to 19% of all subfamilies in the human genome) (Fig. 1a) [1]. In particular, the MER136 subfamily was amongst the top enriched subfamilies, suggesting a potential role in breast cancer transcriptional regulation (Fig. 1b). While DNA transposons are less likely to retain cis-regulatory activity, it has been demonstrated that the insertion of mPing, a MITE in plants, could render neighbouring genes stress-inducible in rice, and this TE-derived regulation is possibly due to the TFBSs present in the MITE [67].

The activity of TEs often occurs at the cost of host genomic stability, as active TEs can generate large mutations or drive unwanted gene expression [24, 32]. The human genome has thus evolved several defence mechanisms against TE activity, one of which is epigenetic suppression via histone tail modifications [25, 26]. As a result, human TEs in somatic tissues are often associated with repressive histone modifications, such as H3K9 methylation [25, 26]. On the other hand, TEs exapted to perform regulatory roles exhibit an active histone modification profile, such as H3K27ac, H3K4me1 and H3K4me3, which is similar to that observed in enhancer or promoter regions [10, 30]. In this study, we examined the histone modification status of the TF-bound TEs and confirmed that TEs contributing TFBSs showed an active epigenetic signature characterised by increased H3K27ac, H3K4me1 and H3K4me3 (Fig. 1c and Additional file 1: Figure S6). H3K4 methylation is often found in both active enhancers and promoters; however, active promoters are also likely to bear other histone modifications, such as increased H3K36me3 [68]. Furthermore, H3K27ac has been established as an epigenetic signature indicating active enhancer elements [49]. We did not find any notable difference in the extent of truncation between bound and unbound elements that may help explain this difference in TF binding (Additional file 1: Figure S7). The co-occurrence of TF binding and active histone modifications indicate a possible co-option of the TF-bound TEs for regulatory roles.

Next, we sought to validate the promoter activity of TF-bound TEs in breast cancer cell lines. The intersection of breast cancer-associated genetic elements with CAGE clusters, TFBSs in MCF7 cells and TEs provided a subset of putative TE-derived promoters. We selected the putative TE-derived promoters of four candidate genes (*SYT1, UCA1, AK4* and *PSAT1*) for in vitro validation. These genes were prioritised based on existing literature describing oncogenic characteristics in various cancers [59–63, 69–77] (summarised in Additional file 4: Table S1). Our filtering process was conservative and required multiple levels of evidence for promoter identification. Final promoter selection was also performed according to existing literature evidence, rather than a prediction of promoter activity. This approach would underestimate the overall contribution of TEs to promoter activity in breast cancer, but would provide disease-relevant candidates for validation.

**Fig. 4:**
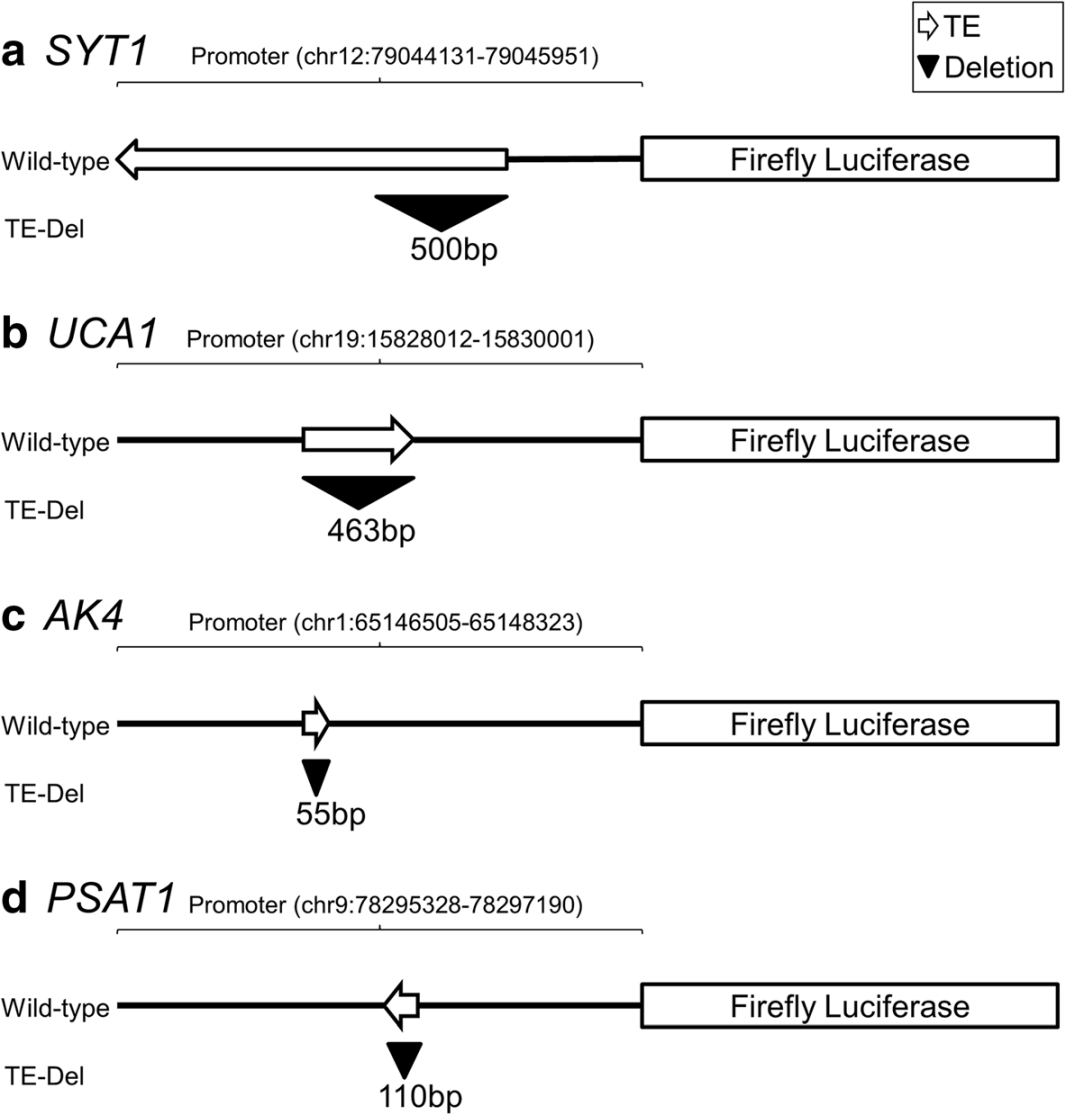
Assembly of the reporter constructs. The wild-type promoters, as well as promoters with the targeted TEs removed (TE-Del), were ligated into the pGL3 Basic vectors, upstream of the luciferase genes. The genomic locations (hg38) of the promoter regions and the sizes of deletions are shown for each of the candidate genes: **a** *SYT1,* **b** *UCA1,* ***c*** *AK4,* **d**) *PSAT1.* Arrows represent TE orientation relative to the candidate genes. Triangles represent the deletions

To evaluate the promoter activity of candidate TEs, the wild-type and TE-deleted promoters were cloned for each gene (Fig. 4). Luciferase assay results indicated that all analysed TEs contributed promoter activity in TNBC cell lines (Fig. 3). In the case of L1PA2-*SYT1*, TE deletion almost completely abolished the promoter activities in two of the three cell lines examined (Fig. 3). Similar results were observed for LTR7C-*UCA1*, while TEs contributed significant promoter activity to *AK4* and *PSAT1* in some, but not all of the cell lines investigated (Fig. 3). Interestingly, we found that the L1PA2-*SYT1* and LTR7C*-UCA1* elements were hypomethylated, and/or more accessible to transcriptional machinery in breast cancer, as indicated by whole-genome methylation capture sequencing and DNase-seq data. The loosening of epigenetic control over these TEs coincided with their strong promoter activities, supporting a link between epigenetic derepression and the regulatory function of individual TEs. The transcriptional activation of the TE-derived promoters potentially led to the aberrant expression of *SYT1* and *UCA1* reported by TCGA [54], as well as activation of their oncogenicity.

The L1PA2-*SYT1* activity was likely driven by the L1 antisense promoter (L1-ASP) located in the first 500 bp of the TE. Similar oncogene activation by L1-ASP has been reported in other cancer types, such as *MET* activation in bladder cancer [35, 78, 79]. *SYT1* is a protein-coding gene found to facilitate the export of the oncogenic growth factor FGF-1 [71]. The promoter activity of L1PA2-*SYT1* in breast cancer cell lines coincided with its increased DNAse sensitivity and decreased DNA methylation, suggesting the L1PA2-*SYT1* element can promote expression of the proto-oncogene *SYT1*. It remains unknown whether this promoter activity is tumour-specific, and whether it contributes to *SYT1* expression under normal physiological conditions. Nonetheless, *SYT1* expression is likely mediated by the L1PA2-*SYT1* transposon in the context of breast cancer cell lines.

The LTR7C-*UCA1* element also contributed essential promoter sequences to the *UCA1* promoter (Fig. 3). This region contains the majority of *UCA1* CAGE tags and the transcription initiation sites of all annotated *UCA1* transcripts, and major peaks of CAGE tags are harboured within the LTR7C element, as shown by the ZENBU genome browser [80] (Additional file 1: Figure S8). Taken together, this suggests that the identified LTR7C element acts as the primary promoter for the *UCA1* oncogene, where the promoter activity is likely driven by the internal promoter within the long terminal repeat. LTR-driven oncogene activation has been reported in multiple cancers, with a notable example in Hodgkin’s lymphoma where an LTR element contributes an alternate promoter causing ectopic activation of the *CSF1R* oncogene [32–34]. Interestingly, *UCA1* encodes an oncogenic lncRNA that inhibits the tumour suppressive miR-143 by direct binding, and also disrupts the translation of tumour suppressor protein p27 through competitive inhibition [59, 60]. *UCA1* expression has been proposed to be a potential biomarker for bladder and pancreatic cancer [61–63]. Confirmation of LTR7C as a critical promoter element for oncogenic lncRNA activation demonstrates the contribution of TE-derived regulatory elements to breast cancer transcriptional regulation.

It is worth noting that many TF-bound TE subfamilies exhibited epigenetic profiles resembling active enhancer regions (Fig. 1c and Additional file 1: Figure S6). Thus, the contribution of TEs to breast cancer transcriptional regulation is likely to be much larger than the few examples highlighted in this study. Further investigation of the regulatory activity and subsequent biological effects of TE-derived enhancers will likely demonstrate extensive exaptation of TEs for oncogene regulation in cancer.

## Conclusions

The exaptation of TEs in the transcriptional regulation of human genes has been demonstrated in an extensive range of biological pathways and cellular functions. TEs serve as a supply of binding sites for RNA polymerases and transcription factor enzymes, and can influence host gene expression by providing promoter or enhancer activities. TEs have also been found to be released from transcriptional repression in several types of epithelial cancer, with specific examples having a direct tumorigenic effect. Here, we have provided a focussed analysis of TE-mediated TF binding and promoter activity in breast cancer cell lines.

We demonstrate that TEs are an abundant source of binding sites for TFs known to show oncogenic activity in breast cancer transcriptional networks. In particular, a number of TE subfamilies were significantly enriched in these TFBSs relative to their genomic occupancy. Within these subfamilies, those bound by TFs were associated with active epigenetic signatures, raising the possibility of these TEs playing a widespread role in breast cancer transcriptional regulation. We subsequently analysed the contribution of TEs to the promoter activities of human genes in three TNBC cell lines. We limited our search to a list of genes identified by the TCGA as being differentially expressed in breast cancer, then focussed on those reported to have oncogenic properties.

While this study is not exhaustive, we have integrated multiple data sources to provide the first evidence for TE-derived transcriptional regulation in breast cancer cell lines. It is likely that more examples will be demonstrated through further study.

## Methods

### Identification of breast cancer-associated TFBSs

To identify TFBSs for C/EBPβ, E2F1 and MYC across the genome, ChIP-seq datasets from MCF7 breast cancer cells were retrieved from GEO and ENCODE in the format of Sequence Read Archive (SRA) and FASTQ respectively [64, 81] (for sources of datasets see Additional file 4: Table S3). Datasets in the SRA format were converted to the FASTQ format with FASTQ-DUMP [82]. The FASTQ sequences (reads) were aligned to the human genome (hg38) via the Burrows-Wheeler Aligner (BWA) program, keeping all possible alignments (−a option) [83]. The mapping of multi-mapped reads were improved using the Multi-Mapper Resolution (MMR) program with 3 iterations [84]. Validation of MMR remapping is provided in Additional file 1: Figure S9. Reads mapping to poorly represented genomic regions (simple repeats, satellite DNA, RNA repeats and low-complexity regions) were removed using BEDTools Intersect (−v option) to avoid skewing of the peak-calling threshold [85].

TF-binding peaks were called using HOMER with a false discovery rate threshold of 0.001 [48]. To increase confidence in peak calling, MYC peaks identified using the ENCODE datasets were filtered using BEDTools Intersect to only retain peaks common to at least two replicates [85]. For MYC, the ChIP-seq datasets from GEO and ENCODE were kept separate for downstream analyses. The genomic locations of TEs (hg38) were obtained from the UCSC RepeatMasker table (including truncated and interrupted elements) [86]. Although the categorisation of TEs is often inconsistent in the literature, here we employ the classification system on the Dfam database [87, 88]. TFBSs within TEs were identified using BEDTools intersect [85].

### Identification of enriched TE subfamilies in TFBSs

For each set of ChIP-seq peaks, the expected representation of each TE subfamily was estimated by the random rotation of the genome and the peak locations (10,000 permutations), then counting ChIP-seq peaks that intersected with a TE using a custom python script. The average count of peaks intersecting with each TE subfamily was divided by the total number of ChIP-seq peaks for each TF to produce the expected possibility of TFBSs being located in TEs by random chance. The observed number of TF peaks overlapping each TE subfamily was counted with no rotation applied. A binomial test was used to identify significantly enriched TE subfamilies. *P*-values were corrected for multiple testing using Bonferroni correction, where *p* < 4.3E-5 indicated statistical significance. Enrichment ratios were calculated for each TE subfamily as the ratios between observed and averaged expected overlaps.

### Epigenetic analyses of TF-bound TEs

To investigate the association between oncogenic TF binding and the epigenetic status of TEs, we analysed the epigenetic profiles of top enriched TEs bound by oncogenic TFs in MCF7 cells using published ChIP-seq datasets (for data sources see Additional file 4: Table S4). The datasets for 6 histone modifications (H3K27ac, H3K4me1, H3K4me3, H3K36me3, H3K9me3 and H3K27me3) were downloaded from ENCODE in FASTQ format, then corrected for irregular sequencing coverage using BBnorm (target = 40, mindepth =1) [89]. Reads were subsequently aligned to the human genome (hg38) with BWA mem, keeping only the primary alignments [83]. MIRb, L2a, AluJb and L2b subfamilies were selected as they were identified to be significantly enriched subfamilies in the oncogenic TFBSs analysed. For each subfamily, TE copies containing a TFBS defined above were identified using BEDTools Intersect, and those without a TFBS were identified using the –v option [85]. A 10 kb region centred on each TE was divided into 50 bp bins. For each histone modification, BEDTools Coverage was used to count reads in each bin, then values were converted to RPKM [85]. RPKM values were subsequently averaged across replicates, normalised to the control by subtraction, and finally averaged across TE copies within each TE subfamily. Plots were smoothed using a Kernel Density Estimation with an Epanechnikov kernel. Histone modification profiles were also plotted for high mapping quality reads (mapQ > = 1 and mapQ > = 10) to confirm observed results were not an artefact of including multi-mapped reads in our analysis (Additional file 1: Figure S10).

We subsequently investigated the degree of truncation for TEs with or without breast cancer-associated TFBSs. For each TE subfamily, the location of each TE, relative to the consensus sequence, was mapped using the “RepStart” and “RepEnd” from RepeatMasker [86]. The coverage of the consensus sequence at 1 bp resolution was calculated for TEs with or without the presence of breast cancer-associated TFBSs, and normalised to the total number of TEs in each group by division.

### Identification of breast cancer-associated genetic elements

A list of ~ 3600 probes identifying differentially expressed genetic elements in breast cancer, previously identified by TCGA [54], was obtained from Professor K. Hoadley (personal communications). To integrate this dataset with hg38, the microarray probe sequences were retrieved from the TCGA website (TCGA Platform Code: AgilentG4502A_07_3), and aligned to the human genome (hg38) using BLASTn (BLASTn parameters: reward = 1; penalty = 3; gap-open = 5; gap-extend = 2; evalue = 3) [54, 55]. Unaligned probes were excluded from further analysis. Probe locations were converted to the BED format using AWK [90].

Genetic elements targeted by the probes were identified by intersecting probe locations with human mRNAs from GENCODE, and lncRNAs from FANTOM CAT and NONCODE, in order of descending priority with BEDTools Intersect (−s and -split options) [85] (for sources of the annotation data see Additional file 4: Table S5). Probes that could not be annotated were discarded.

### Identification of candidate genes with putative TF-bound TE-derived promoters

To increase the likelihood of identifying TE-derived promoters with true transcriptional activity, several filtering steps were used to ensure that the identified TEs contained previously defined TSSs and were located nearby a TFBS defined above. Firstly, the genomic locations of FANTOM CAT CAGE clusters (lv2, permissive) were converted to hg38 by LiftOver [56, 91]. TSSs regulated by breast cancer-associated TFs were identified by intersecting CAGE clusters with TFBSs defined above using BEDTools Window (−w 300) [56, 85]. A 300 bp window was selected to include as many biologically meaningful TSSs as possible for subsequent analysis (Additional file 1: Figure S11). These TSSs were subsequently intersected with TEs using BEDTools Intersect [85]. TSSs located within TEs were then intersected with the promoters of breast cancer-associated genetic elements with BEDTools Intersect (−s option), where the promoters were defined as the 2 kb region around the 5′ ends of the genetic elements [85]. This intersection defined the list of genetic elements for manual curation through literature review of known associations with cancer biology. *SYT1, UCA1, AK4* and *PSAT1* were selected for further analyses.

### Molecular cloning and assembly of reporter constructs

For all molecular cloning, plasmids were purified using QIAprep Spin Miniprep kit (QIAGEN) following the manufacturer’s protocol. Gel extractions were performed using 1% agarose gels, and MinElute Gel Extraction Kit (QIAGEN) following the manufacturer’s protocol, unless otherwise specified.

### Amplification of wild-type promoters

Wild-type promoters were amplified from HeLa genomic DNA by polymerase chain reaction (PCR) using the Kapa HiFi PCR kit (Kapa Biosystem) (for primer sequences see Additional file 4: Table S6). Nested PCR was used for *SYT1*. Amplicons were analysed by agarose gel electrophoresis and extracted.

Amplicons were ligated into the pCR Blunt vector (Thermo Fisher Scientific) using T4 DNA ligase (New England Biolabs) (37 °C for 2–16 h), and transformed into *E.coli* DH5α cells, followed by kanamycin selection and blue-white colony screening. Plasmid sequences were confirmed by capillary sequencing using M13 forward and reverse primers, and customised primers (for primer sequences see Additional file 4: Table S6). Mutation-free clones were identified for all promoters and used for the following steps.

### TE deletion by PCR-driven overlap extension

TEs were deleted from mutation-free wild-type promoters via PCR-driven overlap extension [92] (Fig. 4), using the Kapa HiFi PCR kit (Kapa Biosystem) or Phusion High-Fidelity DNA Polymerase (New England Biolabs). For detail of PCR-driven overlap extension and primer sequences see Additional file 1: Figure S12 and Additional file 4: Table S6 respectively. For *SYT1*, only the first ~ 500 bp of L1PA2 was deleted, as this region was previously shown to be critical for the L1 promoter activities [93] (Fig. 4a).

Final amplicons were ligated into the pCR Blunt vector (Thermo Fisher Scientific) and transformed into *E.coli* DH5α cells. Colonies were screened by colony PCR using MyTaq HS DNA polymerase (Bioline) (for primer sequences see Additional file 4: Table S6). Positive colonies were validated by capillary sequencing, and error-free clones were identified for further use.

### Assembly of reporter constructs

Wild-type and TE-deleted promoters were excised from the pCR Blunt vectors by restriction enzymes digestion (See Additional file 4: Table S6 for insert-enzyme combinations), then ligated into the pGL3 Basic vector (Promega) with T4 DNA ligase (New England Biolabs) (16 °C for 2–16 h), upstream of the firefly luciferase-encoding gene. The ligated plasmids were transformed into *E.coli* DH5α cells, followed by ampicillin selection. Colonies were screened for inserts in the correct orientation via colony PCR using RVprimer3.

### Purification of reporter constructs

*E.coli* DH5α cells were transformed with the reporter constructs, followed by ampicillin selection. For each gene, 3 colonies (clones) per construct were subjected to plasmid extraction. Plasmid DNA of each set of wild-type and TE-deleted constructs, which would be directly compared against each other in luciferase assays, was extracted simultaneously. Plasmid DNA was confirmed for identity and supercoiling by restriction enzyme digestion and gel electrophoresis. Only predominantly supercoiled plasmid DNA was used for transfection.

### Cell culture

TNBC cells lines used in this study were MDA-MB-468, MDA-MB-231 and BT549. All cell lines were cultured in Dulbecco’s Modified Eagle Medium (Thermo Fisher Scientific) supplemented with 10% foetal bovine serum (Thermo Fisher Scientific). Cells were incubated at 37 °C and 5% CO_2_, and passaged with Trypsin-EDTA (Gibco) at 80–90% confluency. Cell passaging at a 1:6 ratio was performed twice a week for MDA-MB-231 cells, and weekly for the other cell lines.

### Transfection of TNBC cells lines

Cells were plated in 24-well plates to give a density of 80–90% at 24 h (plating densities are shown in Additional file 4: Table S7). Triplicate wells were plated for each construct and the positive control. Transfections were performed 24 h post-plating using the Lipofectamine 3000 Reagent (Thermo Fisher Scientific), with equimolar amount of reporter construct (~ 500 ng) and 20 ng of pRL-TK plasmid per well. 500 ng of the pGL3 Promoter vector (Promega) was used as a positive control. Medium was changed at 24 h post-transfection.

### Luciferase assays and statistical analyses

Luciferase activities were measured using the Dual-Glo Luciferase Assay System (Promega). At 48 h post-transfection, medium was removed and cells were lysed using 50 μL of luciferase reagent diluted with 50 μL of PBS. After a 10-min shaking incubation, 90 μL of cell lysate from each well was transferred to a White Opaque 96-well microplate (PerkinElmer). Firefly luminescence was measured at 25 °C on a DTX880 Multimode Detector (Beckman Coulter) (MDA-MB-468 and MDA-MB-231 cells), or CLARIOstar (BMG LABTECH) (BT549 cells). *Renilla* luminescence was measured for each well 10 min after adding 45 μL of Stop&Glo reagents.

The relative luciferase activity of each construct was calculated as the ratio of firefly: *Renilla* luminescence, averaged amongst the triplicates and normalised to the positive control.

For each gene, luciferase assays were replicated in three independent experiments. Relative luciferase activities were normalised to the mean wild-type activity across the replicates for each gene. Using R, the relative luciferase activities of TE-deleted constructs were compared against the wild-type with a one-tailed t-test assuming equal variances, to test our hypothesis that the deletions would reduce promoter activities. *P* < 0.05 indicated statistical significance.

### Bioinformatic analysis of DNA methylation

To investigate the epigenetic status of the candidate TEs in TNBC, their methylation states were evaluated using published whole-genome methylation capture sequencing data [65]. This technique sequences methylated genomic regions by capturing DNA sequences containing methyl-CpGs [65]. This dataset included 6 paired samples. Within each pair, one sample was collected from the TNBC tumour, with another sample collected from the neighbouring normal tissues. The paired sample data were processed as described for TF ChIP-seq data shown above.

As TFBSs were associated with transcriptional activities and thus likely to be epigenetically regulated [94], reads mapping to the previously identified TFBS in each candidate TE were counted using SAMtools, along with reads mapping to the same regions in all TEs from the same subfamily [94]. To normalise for variations in sequencing depth, the methylation level of each candidate TE was calculated as read counts in the TFBS divided by the read count in the same regions of all TEs from the same subfamily. Using R, a one-sample, one-tailed t-test was used to determine whether the difference in the methylation levels between the tumours and the paired normal samples was less than zero. *P* < 0.05 indicated statistical significance.

### Bioinformatic analysis of chromatin accessibility

TE epigenetic states were also evaluated by analysing ENCODE DNase-seq datasets from MCF7 and HMEC (normal breast tissues) cell lines [64] (for DNase-seq data sources see Additional file 4: Table S2). DNase-seq maps accessible chromatin by sequencing DNase I hypersensitive regions, and indirectly reflects epigenetic regulations [95, 96]. Data were analysed as described for TF ChIP-seq data shown above.

Similar to the analysis of DNA methylation, the DNase sensitivity of each candidate TE was calculated as read counts in the TFBS divided by the read count in the same regions in all TEs from the same subfamily, and subsequently averaged within each cell line. Using R, a one-tailed proportion test was employed to determine whether the TEs were more sensitive to DNase cleavage in MCF7 cells relative to HMEC cells. *P* < 0.05 indicated statistical significance.

## Additional files

Additional file 1: Supplementary Figures: contains supplementary figures referenced in the main manuscript (DOCX 3192 kb)

Additional file 2: CAGE clusters and genes mapped to putative TE-derived promoters: contains the list of CAGE clusters and genes overlapping TE-derived promoters (DOCX 54 kb)

Additional file 3: TEs with putative promoter activity: contains the list of genomic locations for TEs with putative promoter activity in breast cancer (XLSX 19 kb)

Additional file 4: Supplementary Tables: contains supplementary tables referenced in the main manuscript (CSV 2 kb)

## Abbreviations

AK4: Adenylate kinase-4
ASP: Antisense promoter
BWA: Burrows-Wheeler Aligner
CAGE: Cap Analysis of Gene Expression
CHD: Chromodomain helicase DNA-binding protein
ChIP-seq: Chromatin immunoprecipitation sequencing
IRF: Interferon regulatory factor
LINE: Long interspersed nuclear element
lncRNA: Long non-coding RNA
LTR: Long terminal repeat
MMR: Multi-Mapper Resolution
PCR: Polymerase chain reaction
PSAT1: Phosphoserine aminotransferase 1
RNA Pol: RNA polymerase
SINE: Short interspersed nuclear element
SRA: Sequence Read Archive
STAT: Signal transducer and activator of transcription
SYT1: Synaptotagmin I
TBP: TATA box binding protein
TCGA: The Cancer Genome Atlas Network
TE: Transposable element
TE-Del: TE-deleted
TF: Transcription factor
TFBS: Transcription factor binding site
TNBC: Triple negative breast cancer
TSS: Transcription start site
UCA1: Urothelial carcinoma associated 1

## References

[1] Lander ES, Linton LM, Birren B, Nusbaum C, Zody MC, Baldwin J (2001). Initial sequencing and analysis of the human genome. Nature..

[2] Chuong EB, Elde NC, Feschotte C (2017). Regulatory activities of transposable elements: from conflicts to benefits. Nat Rev Genet..

[3] McClintock B (1950). The origin and behavior of mutable loci in maize. Proc Natl Acad Sci U S A.

[4] McClintock B (1956). Controlling elements and the gene. Cold Spring Harb Symp Quant Biol.

[5] Orgel LE, Crick FH (1980). Selfish DNA: the ultimate parasite. Nature..

[6] Doolittle WF, Sapienza C (1980). Selfish genes, the phenotype paradigm and genome evolution. Nature..

[7] Ohno S (1972). So much “junk” DNA in our genome. Brookhaven Symp Biol.

[8] Sundaram V, Cheng Y, Ma Z, Li D, Xing X, Edge P (2014). Widespread contribution of transposable elements to the innovation of gene regulatory networks. Genome Res.

[9] van de Lagemaat LN, Landry J-R, Mager DL, Medstrand P (2003). Transposable elements in mammals promote regulatory variation and diversification of genes with specialized functions. Trends Genet.

[10] Chuong EB, Elde NC, Feschotte C (2016). Regulatory evolution of innate immunity through co-option of endogenous retroviruses. Science..

[11] Feschotte C (2008). Transposable elements and the evolution of regulatory networks. Nat Rev Genet..

[12] Emera D, Casola C, Lynch VJ, Wildman DE, Agnew D, Wagner GP (2012). Convergent evolution of endometrial prolactin expression in primates, mice, and elephants through the independent recruitment of transposable elements. Mol Biol Evol.

[13] Romanish MT, Lock WM, van de Lagemaat LN, Dunn CA, Mager DL (2007). Repeated recruitment of LTR retrotransposons as promoters by the anti-apoptotic locus NAIP during mammalian evolution. PLoS Genet.

[14] Lunyak VV, Prefontaine GG, Núñez E, Cramer T, Ju B-G, Ohgi KA (2007). Developmentally regulated activation of a SINE B2 repeat as a domain boundary in organogenesis. Science..

[15] Jordan IK, Rogozin IB, Glazko GV, Koonin EV (2003). Origin of a substantial fraction of human regulatory sequences from transposable elements. Trends Genet.

[16] Cruickshanks HA, Tufarelli C (2009). Isolation of cancer-specific chimeric transcripts induced by hypomethylation of the LINE-1 antisense promoter. Genomics..

[17] Gifford WD, Pfaff SL, Macfarlan TS (2013). Transposable elements as genetic regulatory substrates in early development. Trends Cell Biol.

[18] Faulkner GJ, Kimura Y, Daub CO, Wani S, Plessy C, Irvine KM (2009). The regulated retrotransposon transcriptome of mammalian cells. Nat Genet.

[19] Simonti CN, Pavlicev M, Capra JA (2017). Transposable element exaptation into regulatory regions is rare, influenced by evolutionary age, and subject to pleiotropic constraints. Mol Biol Evol.

[20] Kanamori-Katayama M, Itoh M, Kawaji H, Lassmann T, Katayama S, Kojima M (2011). Unamplified cap analysis of gene expression on a single-molecule sequencer. Genome Res.

[21] Pavlicev M, Hiratsuka K, Swaggart KA, Dunn C, Muglia L (2015). Detecting endogenous retrovirus-driven tissue-specific gene transcription. Genome Biol Evol.

[22] Cohen CJ, Lock WM, Mager DL (2009). Endogenous retroviral LTRs as promoters for human genes: a critical assessment. Gene..

[23] Huda A, Jordan IK (2009). Epigenetic regulation of mammalian genomes by transposable elements. Ann N Y Acad Sci.

[24] Slotkin RK, Martienssen R (2007). Transposable elements and the epigenetic regulation of the genome. Nat Rev Genet..

[25] Huda A, Mariño-Ramírez L, Jordan IK (2010). Epigenetic histone modifications of human transposable elements: genome defense versus exaptation. Mob DNA.

[26] Kondo Y, Issa JP (2003). Enrichment for histone H3 lysine 9 methylation at Alu repeats in human cells. J Biol Chem.

[27] Yoder JA, Walsh CP, Bestor TH (1997). Cytosine methylation and the ecology of intragenomic parasites. Trends Genet.

[28] Lee E, Iskow R, Yang L, Gokcumen O, Haseley P, Luquette LJ (2012). Landscape of somatic retrotransposition in human cancers. Science..

[29] Handy DE, Castro R, Loscalzo J (2011). Epigenetic modifications: basic mechanisms and role in cardiovascular disease. Circulation..

[30] Huda A, Tyagi E, Marino-Ramirez L, Bowen NJ, Jjingo D, Jordan IK (2011). Prediction of transposable element derived enhancers using chromatin modification profiles. PLoS One.

[31] Choi SH, Worswick S, Byun HM, Shear T, Soussa JC, Wolff EM (2009). Changes in DNA methylation of tandem DNA repeats are different from interspersed repeats in cancer. Int J Cancer.

[32] Babaian A, Mager DL (2016). Endogenous retroviral promoter exaptation in human cancer. Mob DNA.

[33] Anwar Sumadi, Wulaningsih Wahyu, Lehmann Ulrich (2017). Transposable Elements in Human Cancer: Causes and Consequences of Deregulation. International Journal of Molecular Sciences.

[34] Lamprecht B, Walter K, Kreher S, Kumar R, Hummel M, Lenze D (2010). Derepression of an endogenous long terminal repeat activates the CSF1R proto-oncogene in human lymphoma. Nat Med.

[35] Wolff EM, Byun H-M, Han HF, Sharma S, Nichols PW, Siegmund KD (2010). Hypomethylation of a LINE-1 promoter activates an alternate transcript of the MET oncogene in bladders with cancer. PLoS Genet.

[36] Lock FE, Rebollo R, Miceli-Royer K, Gagnier L, Kuah S, Babaian A (2014). Distinct isoform of FABP7 revealed by screening for retroelement-activated genes in diffuse large B-cell lymphoma. Proc Natl Acad Sci U S A.

[37] Knudsen ES, McClendon AK, Franco J, Ertel A, Fortina P, Witkiewicz AK (2015). RB loss contributes to aggressive tumor phenotypes in MYC-driven triple negative breast cancer. Cell Cycle.

[38] Kumar P, Aggarwal R (2016). An overview of triple-negative breast cancer. Arch Gynecol Obstet.

[39] Engebraaten O, Vollan HKM, Børresen-Dale A-L (2013). Triple-negative breast cancer and the need for new therapeutic targets. Am J Pathol.

[40] De Ruijter TC, Veeck J, de Hoon JPJ, van Engeland M, Tjan-Heijnen VC (2011). Characteristics of triple-negative breast cancer. J Cancer Res Clin Oncol.

[41] Abreu MM, Sealy L (2010). The C/EBPbeta isoform, liver inhibitory protein (LIP), induces autophagy in breast cancer cell lines. Exp Cell Res.

[42] Camarda R, Zhou Z, Kohnz RA, Balakrishnan S, Mahieu C, Anderton B (2016). Inhibition of fatty acid oxidation as a therapy for MYC-overexpressing triple-negative breast cancer. Nat Med.

[43] Horiuchi D, Kusdra L, Huskey NE, Chandriani S, Lenburg ME, Gonzalez-Angulo AM (2012). MYC pathway activation in triple-negative breast cancer is synthetic lethal with CDK inhibition. J Exp Med.

[44] Janghorban M, Farrell AS, Allen-Petersen BL, Pelz C, Daniel CJ, Oddo J (2014). Targeting c-MYC by antagonizing PP2A inhibitors in breast cancer. Proc Natl Acad Sci U S A.

[45] Zacharatos P, Kotsinas A, Evangelou K, Karakaidos P, Vassiliou L-V, Rezaei N (2004). Distinct expression patterns of the transcription factor E2F-1 in relation to tumour growth parameters in common human carcinomas. J Pathol.

[46] Gomis RR, Alarcon C, Nadal C, Van Poznak C, Massague J (2006). C/EBPbeta at the core of the TGFbeta cytostatic response and its evasion in metastatic breast cancer cells. Cancer Cell.

[47] Milde-Langosch K, Loning T, Bamberger AM (2003). Expression of the CCAAT/enhancer-binding proteins C/EBPalpha, C/EBPbeta and C/EBPdelta in breast cancer: correlations with clinicopathologic parameters and cell-cycle regulatory proteins. Breast Cancer Res Treat.

[48] Heinz S, Benner C, Spann N, Bertolino E, Lin YC, Laslo P (2010). Simple combinations of lineage-determining transcription factors prime cis-regulatory elements required for macrophage and B cell identities. Mol Cell.

[49] Creyghton MP, Cheng AW, Welstead GG, Kooistra T, Carey BW, Steine EJ (2010). Histone H3K27ac separates active from poised enhancers and predicts developmental state. Proc Natl Acad Sci U S A.

[50] Zentner GE, Tesar PJ, Scacheri PC (2011). Epigenetic signatures distinguish multiple classes of enhancers with distinct cellular functions. Genome Res.

[51] Heintzman ND, Stuart RK, Hon G, Fu Y, Ching CW, Hawkins RD (2007). Distinct and predictive chromatin signatures of transcriptional promoters and enhancers in the human genome. Nat Genet.

[52] Boyer LA, Plath K, Zeitlinger J, Brambrink T, Medeiros LA, Lee TI (2006). Polycomb complexes repress developmental regulators in murine embryonic stem cells. Nature..

[53] Peters AH, Kubicek S, Mechtler K, O’Sullivan RJ, Derijck AA, Perez-Burgos L (2003). Partitioning and plasticity of repressive histone methylation states in mammalian chromatin. Mol Cell.

[54] Cancer Genome Atlas Network (2012). Comprehensive molecular portraits of human breast tumours. Nature..

[55] Altschul SF, Gish W, Miller W, Myers EW, Lipman DJ (1990). Basic local alignment search tool. J Mol Biol.

[56] Hon C-C, Ramilowski JA, Harshbarger J, Bertin N, Rackham OJL, Gough J (2017). An atlas of human long non-coding RNAs with accurate 5′ ends. Nature..

[57] Zhao Y, Li H, Fang S, Kang Y, Wu W, Hao Y (2016). NONCODE 2016: an informative and valuable data source of long non-coding RNAs. Nucleic Acids Res.

[58] Harrow J, Frankish A, Gonzalez JM, Tapanari E, Diekhans M, Kokocinski F (2012). GENCODE: the reference human genome annotation for the ENCODE project. Genome Res.

[59] Huang J, Zhou N, Watabe K, Lu Z, Wu F, Xu M (2014). Long non-coding RNA UCA1 promotes breast tumor growth by suppression of p27 (Kip1). Cell Death Dis.

[60] Tuo YL, Li XM, Luo J (2015). Long noncoding RNA UCA1 modulates breast cancer cell growth and apoptosis through decreasing tumor suppressive miR-143. Eur Rev Med Pharmacol Sci.

[61] Chen P, Wan D, Zheng D, Zheng Q, Wu F, Zhi Q (2016). Long non-coding RNA UCA1 promotes the tumorigenesis in pancreatic cancer. Biomed Pharmacother.

[62] H-h H, L-k H, Pan X, Wu C-Y, Huang H, Li B (2016). Long non-coding RNA UCA1 is a predictive biomarker of cancer. Oncotarget..

[63] Wang X-S, Zhang Z, Wang H-C, Cai J-L, Xu Q-W, Li M-Q (2006). Rapid identification of UCA1 as a very sensitive and specific unique marker for human bladder carcinoma. Clin Cancer Res.

[64] ENCODE Project Consortium (2012). An integrated encyclopedia of DNA elements in the human genome. Nature..

[65] Stirzaker C, Zotenko E, Song JZ, Qu W, Nair SS, Locke WJ (2015). Methylome sequencing in triple-negative breast cancer reveals distinct methylation clusters with prognostic value. Nat Commun.

[66] Feschotte C, Zhang X, Wessler SR. Miniature inverted-repeat transposable elements (MITEs) and their relationship with established DNA transposons. In: Craig N, Craigie R, Gellert M, Lambowitz A, editors. Mobile DNA II. Washington, D.C: American Society of Microbiology; 2002. p. 1147–1158.

[67] Naito K, Zhang F, Tsukiyama T, Saito H, Hancock CN, Richardson AO (2009). Unexpected consequences of a sudden and massive transposon amplification on rice gene expression. Nature..

[68] Barski A, Cuddapah S, Cui K, Roh T-Y, Schones DE, Wang Z (2007). High-resolution profiling of histone methylations in the human genome. Cell..

[69] Lai Y, Lou X, Diao J, Shin Y-K (2015). Molecular origins of synaptotagmin 1 activities on vesicle docking and fusion pore opening. Sci Rep.

[70] Sudhof TC (2013). A molecular machine for neurotransmitter release: synaptotagmin and beyond. Nat Med.

[71] Egan JB, Barrett MT, Champion MD, Middha S, Lenkiewicz E, Evers L (2014). Whole genome analyses of a well-differentiated liposarcoma reveals novel SYT1 and DDR2 rearrangements. PLoS One.

[72] Zhang S, Dong X, Ji T, Chen G, Shan L (2017). Long non-coding RNA UCA1 promotes cell progression by acting as a competing endogenous RNA of ATF2 in prostate cancer. Am J Transl Res.

[73] Zhen S, Hua L, Liu YH, Sun XM, Jiang MM, Chen W (2017). Inhibition of long non-coding RNA UCA1 by CRISPR/Cas9 attenuated malignant phenotypes of bladder cancer. Oncotarget..

[74] Fujisawa K, Terai S, Takami T, Yamamoto N, Yamasaki T, Matsumoto T (2016). Modulation of anti-cancer drug sensitivity through the regulation of mitochondrial activity by adenylate kinase 4. J Exp Clin Cancer Res.

[75] Jan YH, Tsai HY, Yang CJ, Huang MS, Yang YF, Lai TC (2012). Adenylate kinase-4 is a marker of poor clinical outcomes that promotes metastasis of lung cancer by downregulating the transcription factor ATF3. Cancer Res.

[76] Liao KM, Chao TB, Tian YF, Lin CY, Lee SW, Chuang HY (2016). Overexpression of the PSAT1 gene in nasopharyngeal carcinoma is an indicator of poor prognosis. J Cancer.

[77] Liu B, Jia Y, Cao Y, Wu S, Jiang H, Sun X (2016). Overexpression of phosphoserine aminotransferase 1 (PSAT1) predicts poor prognosis and associates with tumor progression in human esophageal squamous cell carcinoma. Cell Physiol Biochem.

[78] Mätlik K, Redik K, Speek M (2006). L1 antisense promoter drives tissue-specific transcription of human genes. J Biomed Biotechnol.

[79] Xiao-Jie L, Hui-Ying X, Qi X, Jiang X, Shi-Jie M (2016). LINE-1 in cancer: multifaceted functions and potential clinical implications. Genet Med.

[80] Severin J, Lizio M, Harshbarger J, Kawaji H, Daub CO, Hayashizaki Y (2014). Interactive visualization and analysis of large-scale sequencing datasets using ZENBU. Nat Biotechnol.

[81] Barrett T, Wilhite SE, Ledoux P, Evangelista C, Kim IF, Tomashevsky M (2013). NCBI GEO: archive for functional genomics data sets—update. Nucleic Acids Res.

[82] National Center for Biotechnology Information (US). Download Guide [Updated 2016 Jan 14]. In: SRA Handbook [Internet]. Bethesda (MD): National Center for Biotechnology Information (US); 2009. Available from: https://www.ncbi.nlm.nih.gov/books/NBK242621/.

[83] Li H, Durbin R (2009). Fast and accurate short read alignment with burrows–wheeler transform. Bioinformatics..

[84] Kahles A, Behr J, Rätsch G (2016). MMR: a tool for read multi-mapper resolution. Bioinformatics..

[85] Quinlan AR, Hall IM (2010). BEDTools: a flexible suite of utilities for comparing genomic features. Bioinformatics..

[86] Smit A, Hubley, R & Green, P. RepeatMasker Open-4.0. 2013-2015. http://www.repeatmasker.org. Accessed 02 Apr 2017.

[87] Hubley R, Finn RD, Clements J, Eddy SR, Jones TA, Bao W (2016). The Dfam database of repetitive DNA families. Nucleic Acids Res.

[88] Kapitonov VV, Jurka J (2008). A universal classification of eukaryotic transposable elements implemented in Repbase. Nat Rev Genet..

[89] Bushnell B. BBMap short read aligner, and other bioinformatic tools. 2015. https://sourceforge.net/projects/bbmap/. Accessed 23 Mar 2018.

[90] Aho AV, Kernighan BW, Weinberger PJ (1979). Awk - a pattern scanning and processing language. Softw Pract Exp.

[91] Hinrichs AS, Karolchik D, Baertsch R, Barber GP, Bejerano G, Clawson H (2006). The UCSC genome browser database: update 2006. Nucleic Acids Res.

[92] Heckman KL, Pease LR (2007). Gene splicing and mutagenesis by PCR-driven overlap extension. Nat Protoc.

[93] Speek M (2001). Antisense promoter of human L1 retrotransposon drives transcription of adjacent cellular genes. Mol Cell Biol.

[94] Li H, Handsaker B, Wysoker A, Fennell T, Ruan J, Homer N (2009). The sequence alignment/map format and SAMtools. Bioinformatics..

[95] Meyer CA, Liu XS (2014). Identifying and mitigating bias in next-generation sequencing methods for chromatin biology. Nat Rev Genet.

[96] Tsompana M, Buck MJ (2014). Chromatin accessibility: a window into the genome. Epigenetics Chromatin.

