# Supplementary material for: Human transposons are an abundant supply of transcription factor binding sites and promoter activities in breast cancer cell lines
Source: Mob DNA. 2019 Apr 27;10:16. doi: 10.1186/s13100-019-0158-3 (PMC6486989; doi:10.1186/s13100-019-0158-3)
Supplement: Supplementary file 1 — Supplementary Figures: contains supplementary figures referenced in the main manuscript (DOCX 3192 kb) [file 13100_2019_158_MOESM1_ESM.docx]

# Supplementary Figures

**
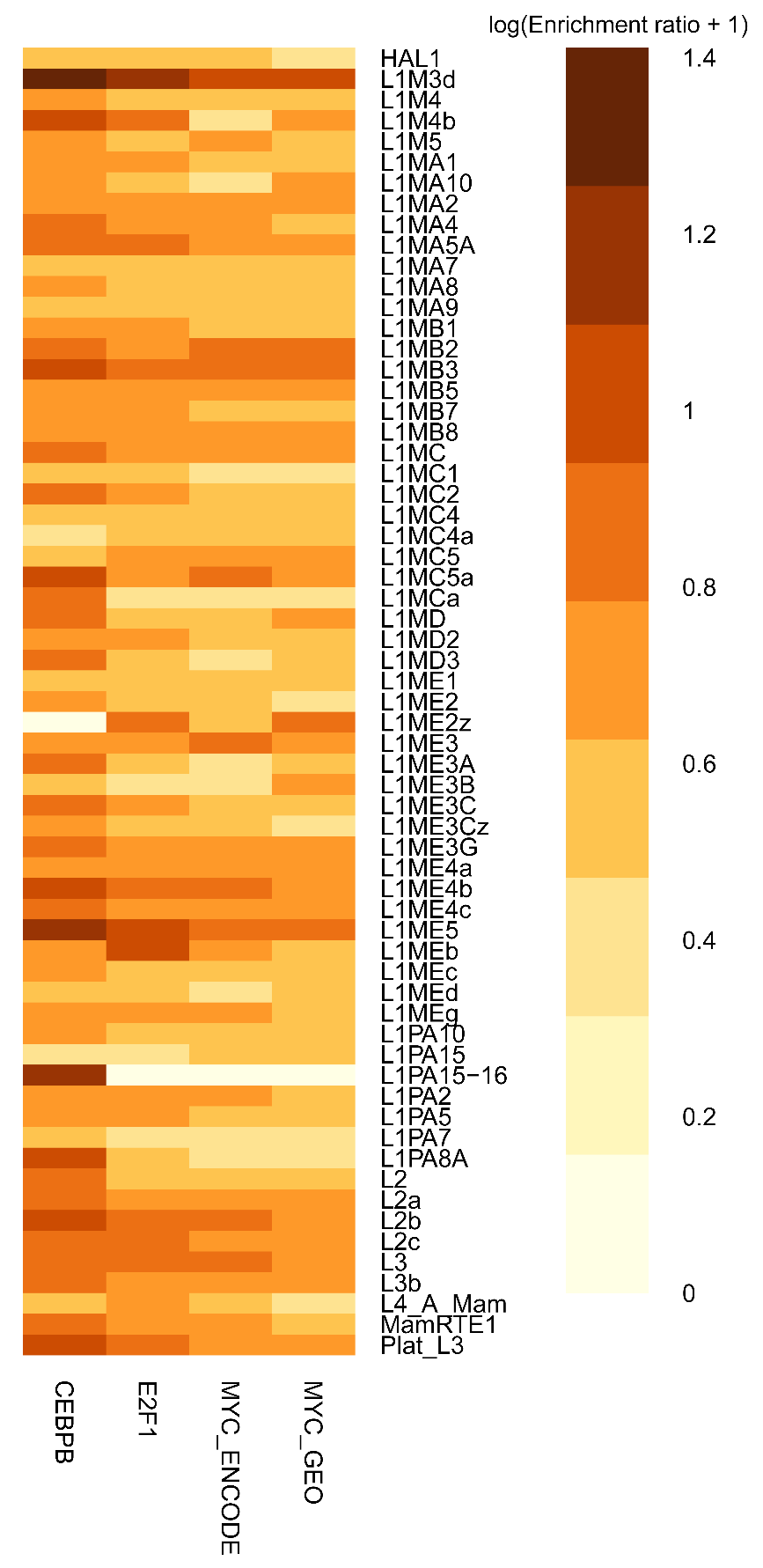
**

**Supplementary Fig.S1.** LINE subfamilies identified to be significantly enriched in breast cancer-associated TFBSs in MCF7 cells. The enrichment ratios (log(x+1)) are shown in the heatmap.

**
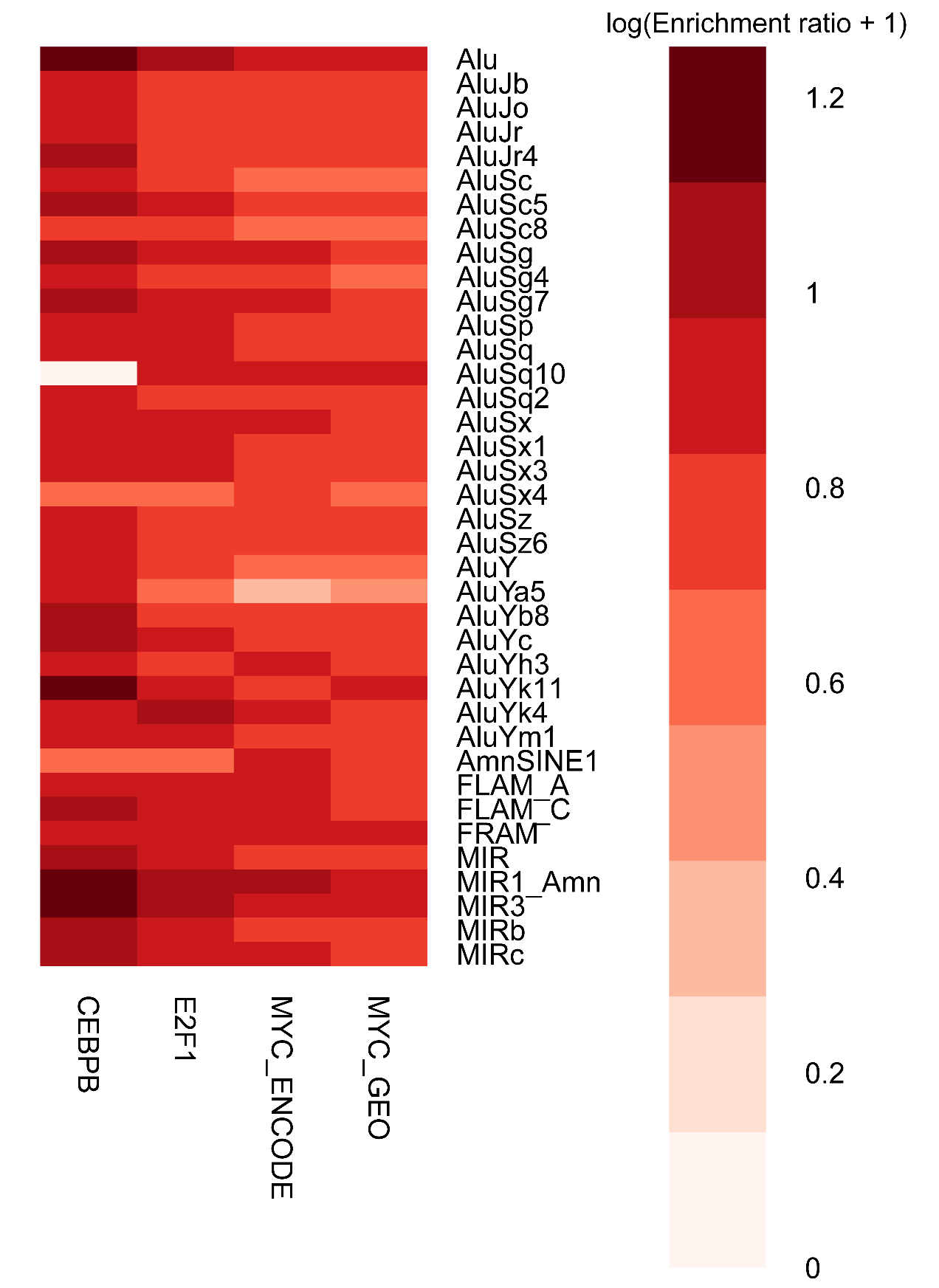
**

**Supplementary Fig.S2.** SINE subfamilies identified to be significantly enriched in breast cancer-associated TFBSs in MCF7 cells. The enrichment ratios (log(x+1)) are shown in the heatmap.

**
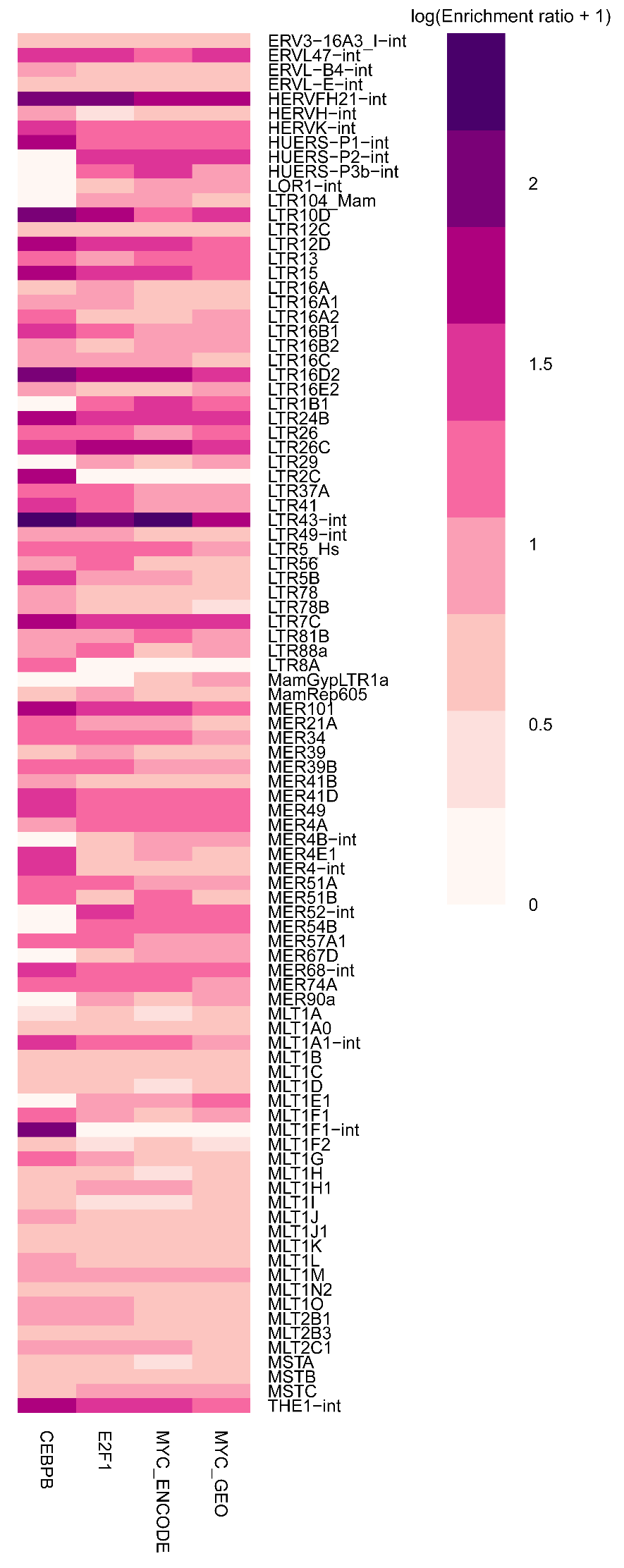
**

**Supplementary Fig.S3.** LTR subfamilies identified to be significantly enriched in breast cancer-associated TFBSs in MCF7 cells. The enrichment ratios (log(x+1)) are shown in the heatmap.

**
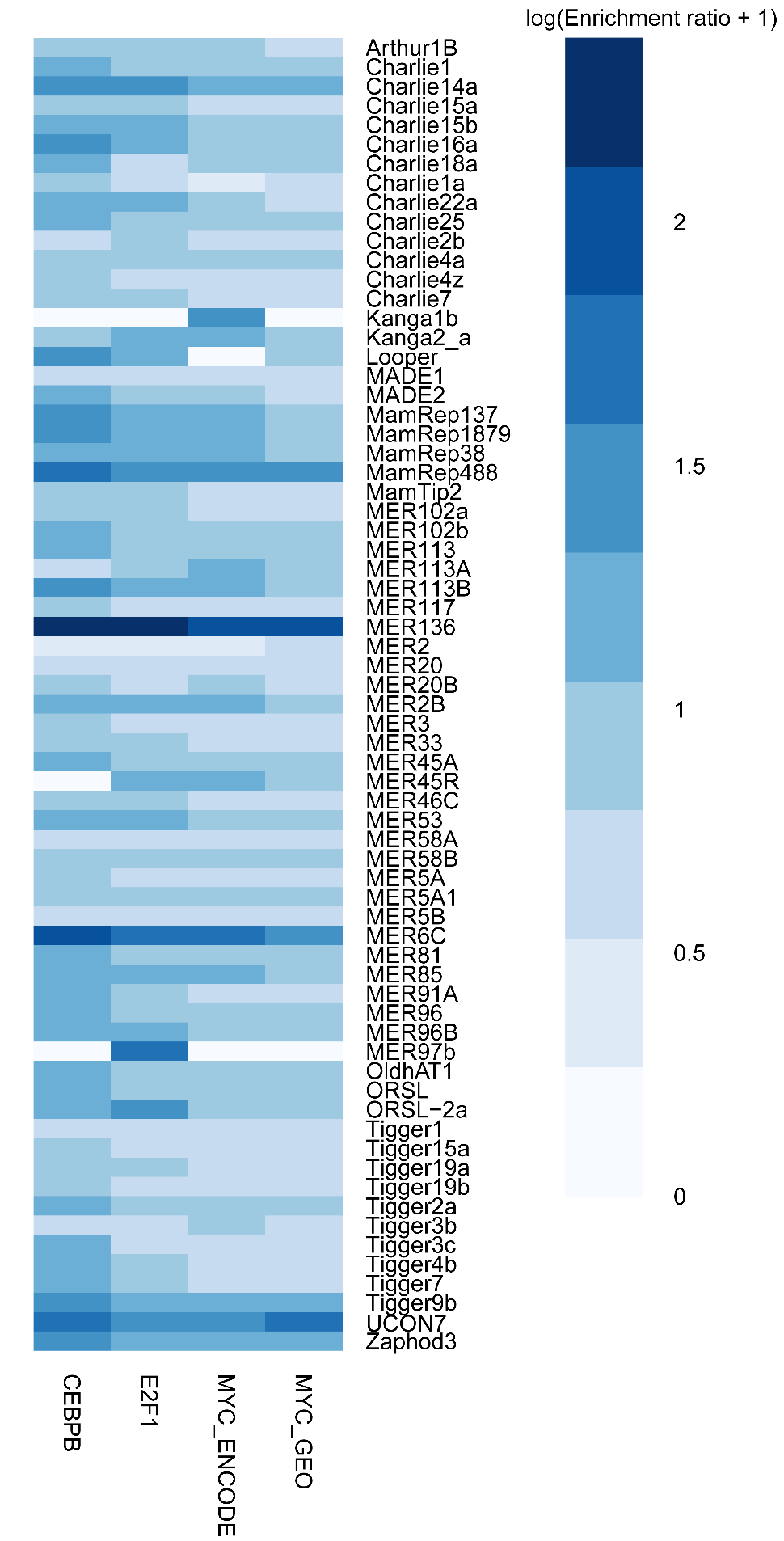
**

**Supplementary Fig.S4.** DNA transposon subfamilies identified to be significantly enriched in breast cancer-associated TFBSs in MCF7 cells. The enrichment ratios (log(x+1)) are shown in the heatmap.

**
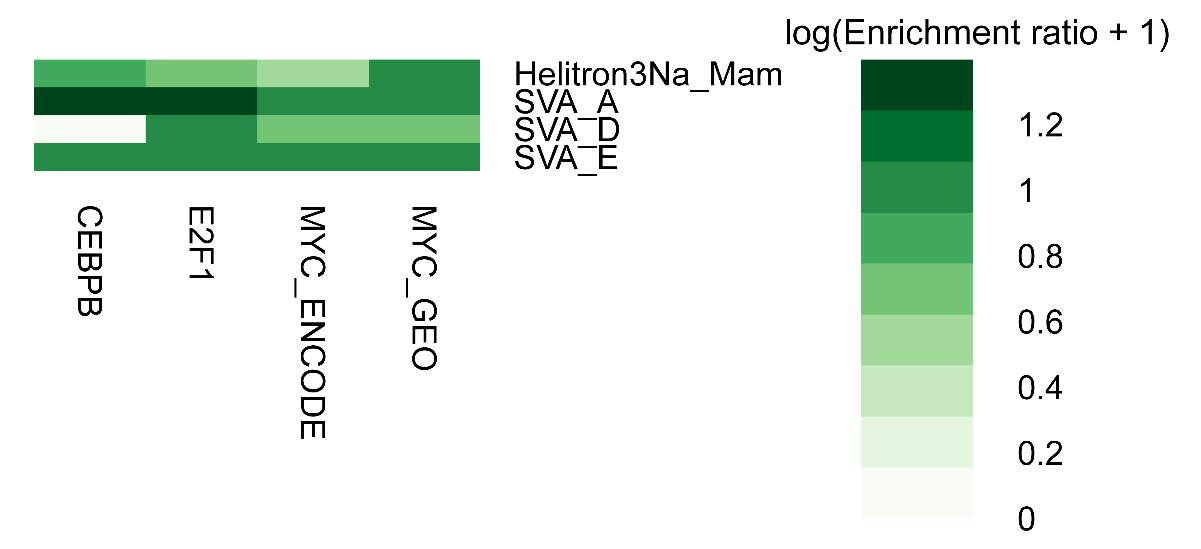
**

**Supplementary Fig.S5.** Other transposon subfamilies identified to be significantly enriched in breast cancer-associated TFBSs in MCF7 cells. The enrichment ratios (log(x+1)) are shown in the heatmap.


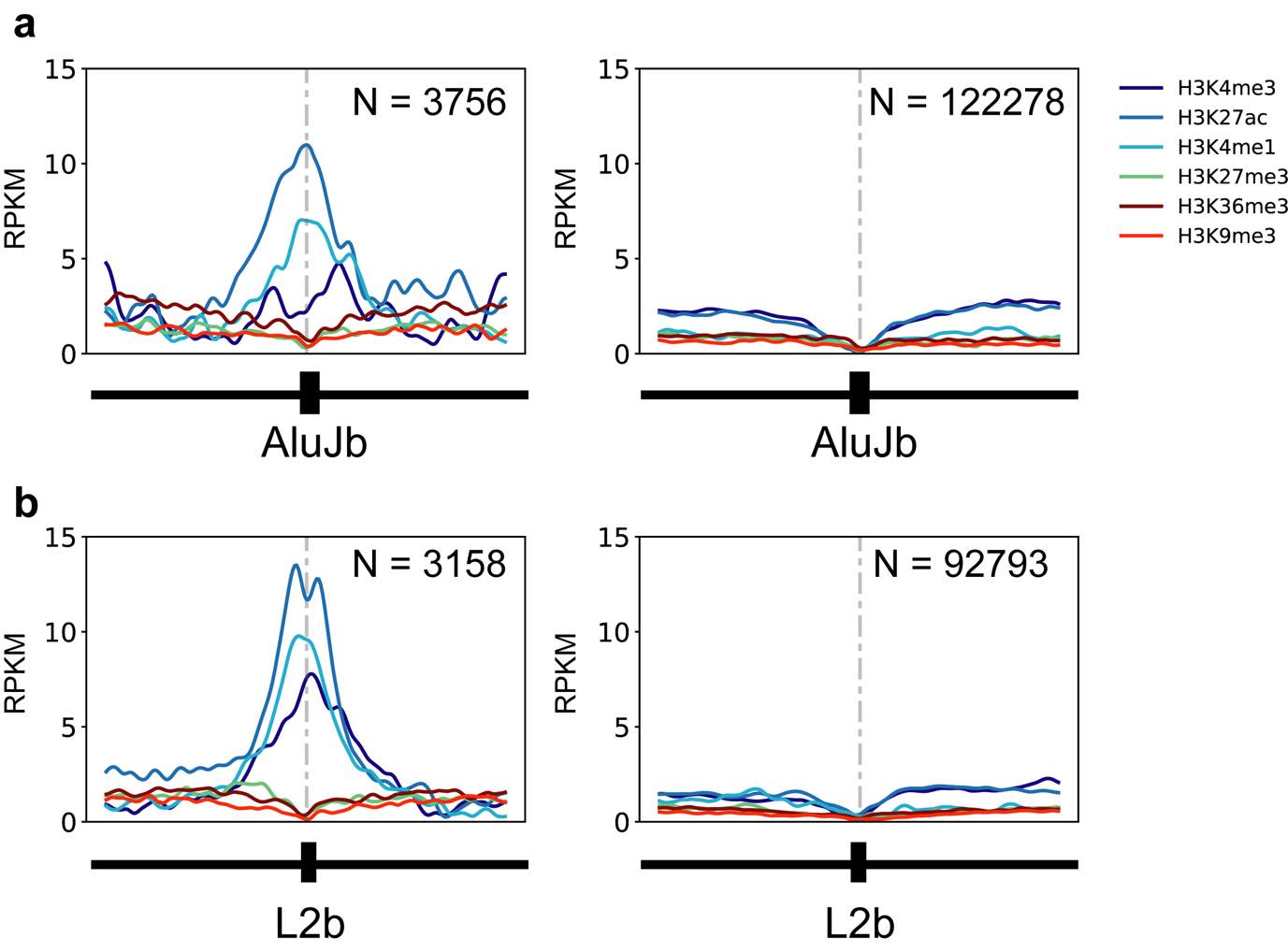


**Supplementary Fig.S6.** Histone modification profile of TF-bound TEs in MCF7 cells. Subfamilies enriched in the binding sites of E2F1, MYC and C/EBPβ were investigated for their association with histone marks using published ChIP-seq datasets. The left panels display the histone modification profile of the TE copies containing TFBSs, while the right panels display the profile of TE copies lacking TFBSs. The average RPKM values at 50bp resolution over a 10kb region centred on the TEs are plotted for **a**) AluJb and **b**) L2b.


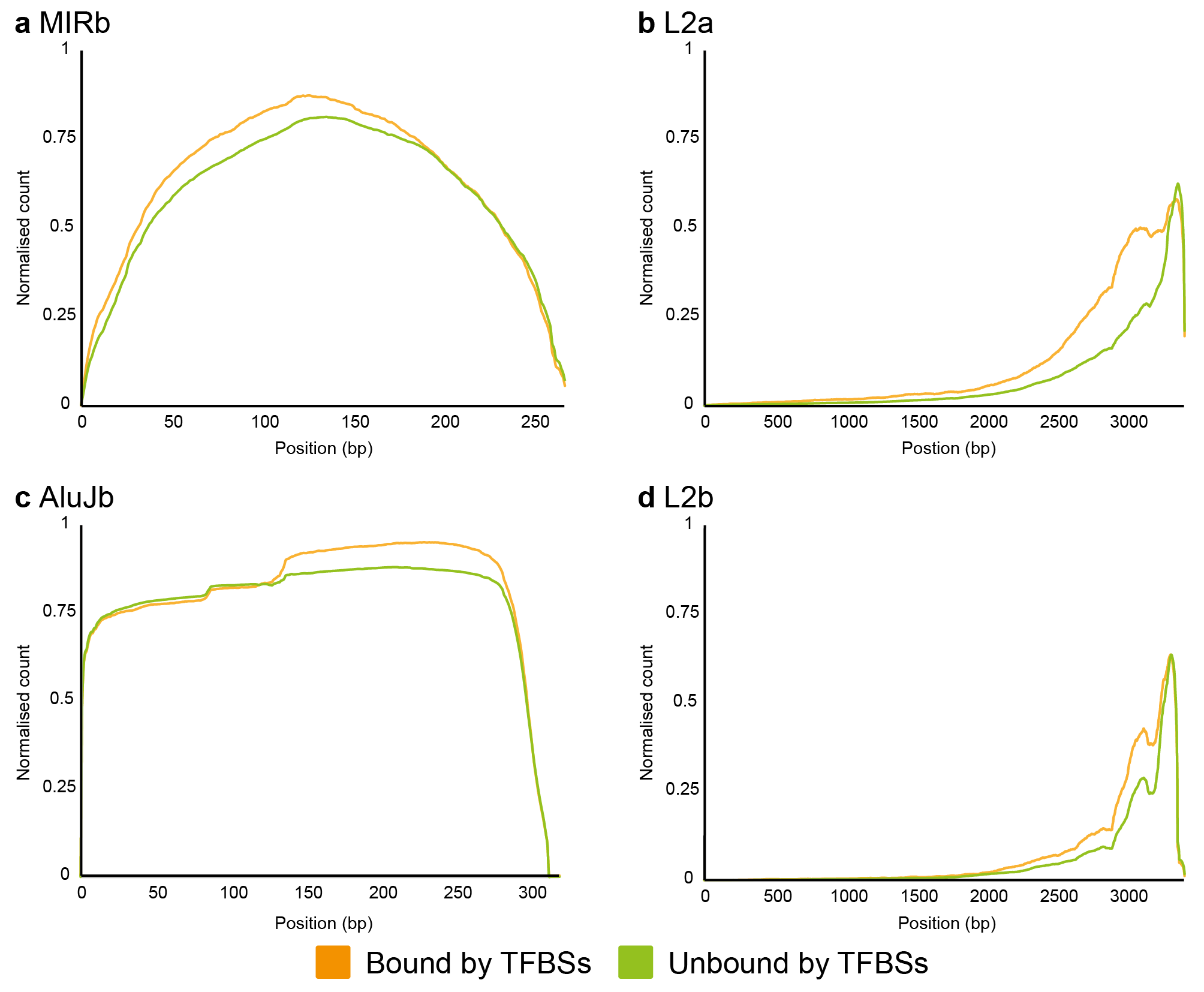


**Supplementary Fig.S7.** The extent of truncation of TEs bound or unbound by breast cancer-associated TFs. For each subfamily, the locations of TEs, relative to the consensus sequence, were mapped using the “RepStart” and “RepEnd” from RepeatMasker [1]. The coverage of the consensus sequence at 1bp resolution was calculated for TEs with (orange) or without (green) the presence of breast cancer-associated TFBSs, and normalised to the total number of TEs in each group by division. The coverage was calculated for **a**) MIRb, **b**) L2a, **c**) AluJb and **d**) L2b.


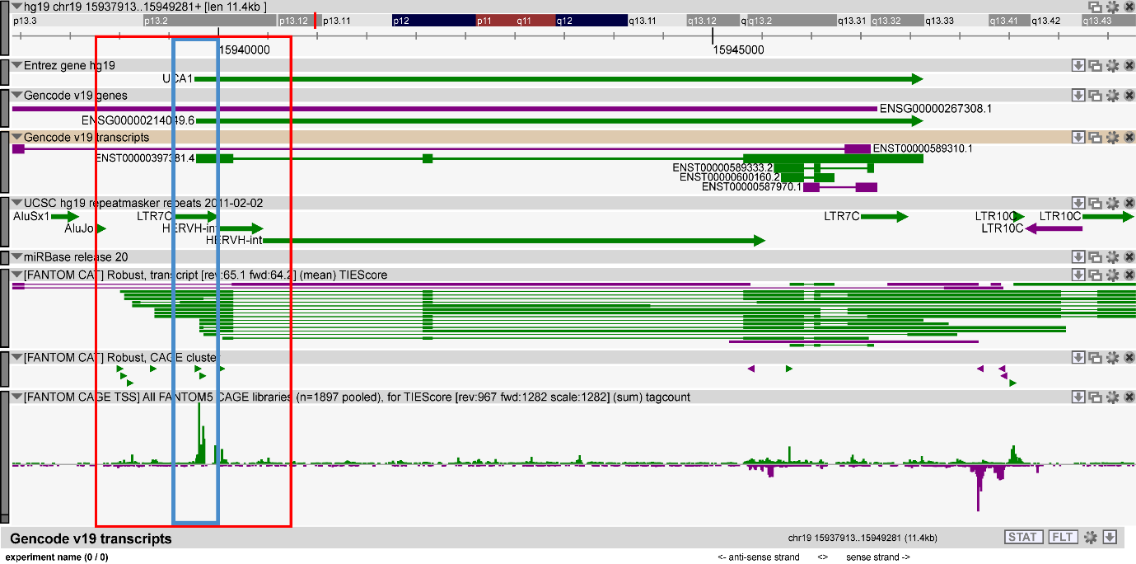


**Supplementary Fig.S8.** The ZENBU genome browser (hg19) view of LTR7C-*UCA1* [2]*.* The promoter region cloned for luciferase assays (red) contained the majority of *UCA1* CAGE clusters and the transcription initiation sites for most *UCA1* transcripts. The LTR7C-*UCA1* element (blue) overlapped with dominant CAGE cluster peaks.

**
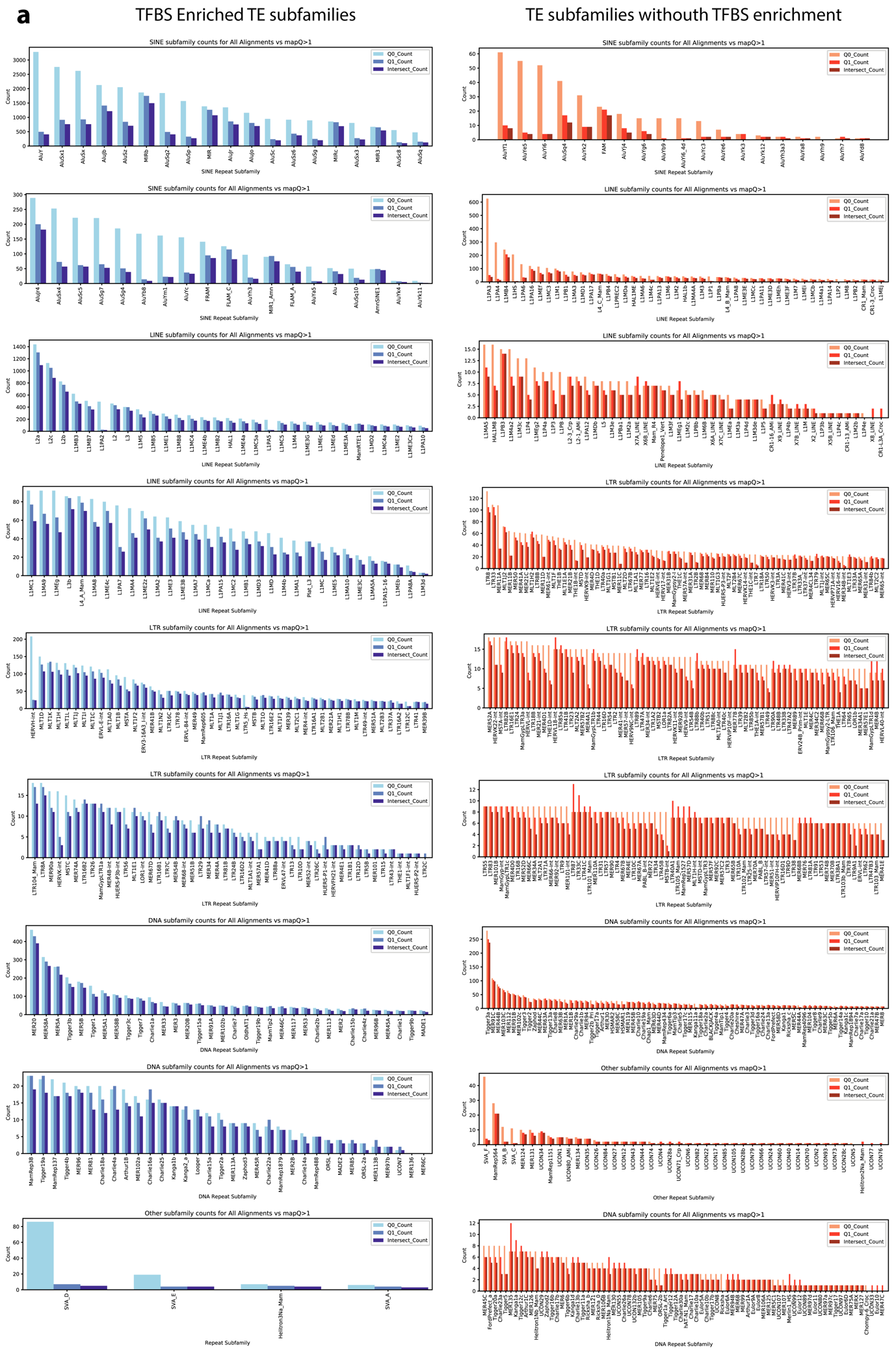
**

**
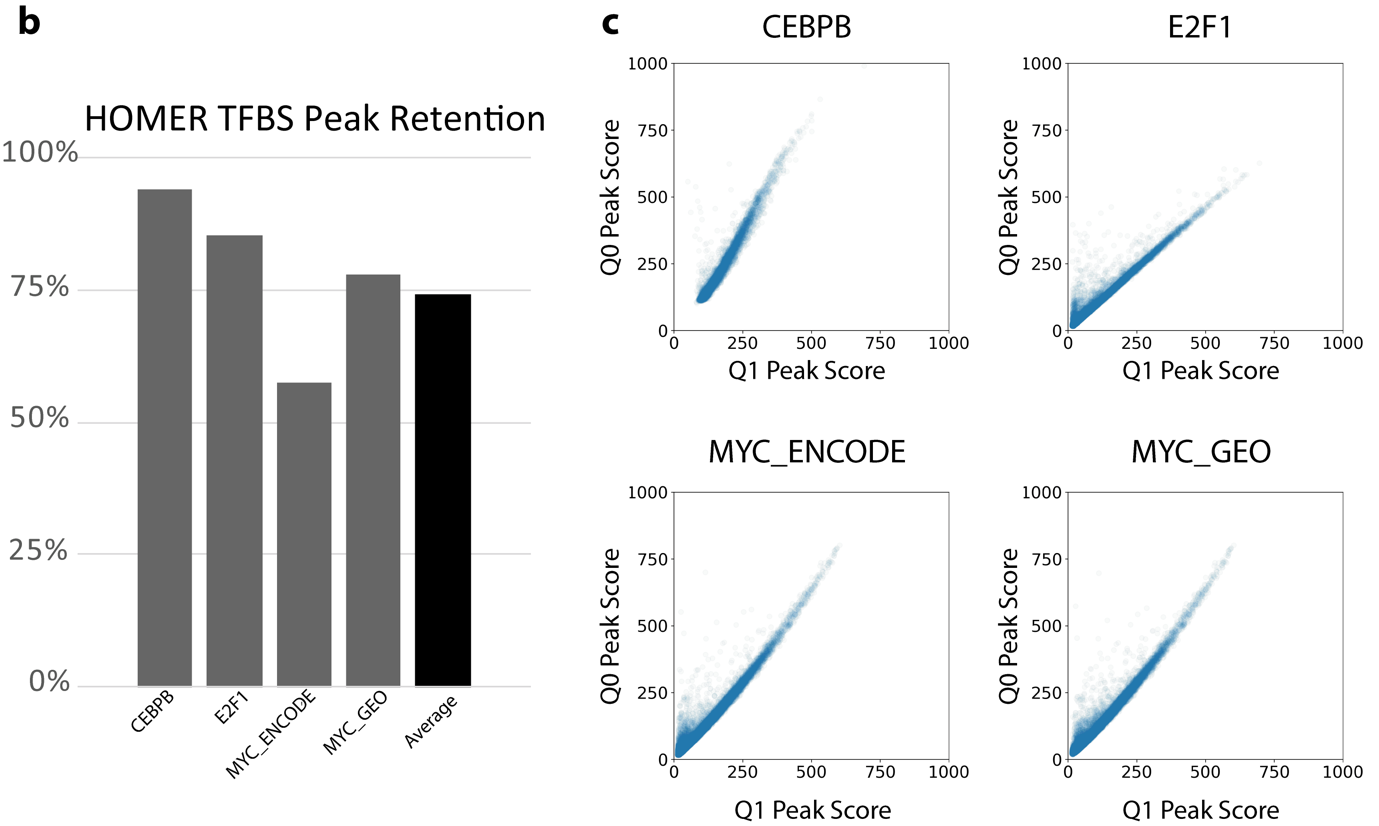
**

**Supplementary Fig.S9.** Validation of MMR remapping of TF ChIP-seq data [3]. TF binding peaks were called with HOMER using either all primary alignments following MMR processing (Q0), or primary alignments with low mapping quality reads removed (Q1) (mapQ >=1 cutoff) [4]. To Investigate whether TF binding peaks within TEs would still be detected if multi-mapping reads were excluded, we plotted the number of peaks identified within each repeat subfamily in Q0 peaks (Q0 counts), Q1 peaks (Q1 counts) or in both Q0 and Q1 peaks (Intersect counts) (**a**). Subfamilies identified as being enriched for TF binding peaks are shown in blue in the left column of plots and TE subfamilies without TF binding peak enrichment are shown in red in the right-hand column of plots. Counts for both Q1 and Q0 peaks are overall very similar, though more TFBSs are identified within TE by including all primary alignments. A few subfamilies with massively increased peak counts were found in both enriched and non-enriched subfamilies. To confirm that inclusion of multi-mapped reads did not affect overall peak calling, we compared the retention of Q1 peaks in the Q0 peak set for each TF (**b**). Peak retention was variable and generally high with the exception of the MYC_ENCODE dataset. An average retention of ~75% was observed across all datasets. Finally we compared the scores assigned to each of the peaks that were common to both Q0 and Q1 peak sets (**c**). Peak scores were again well conserved between Q0 and Q1, indicated by the strong diagonal trend. A noticeable skew toward the top left is observed for all TF data-sets indicating that some low score Q1 peaks receive higher scores when all reads are considered. Overall, this data suggests that MMR is suitable for the processing of multimapping reads in ChIP-seq type sequencing data.

**
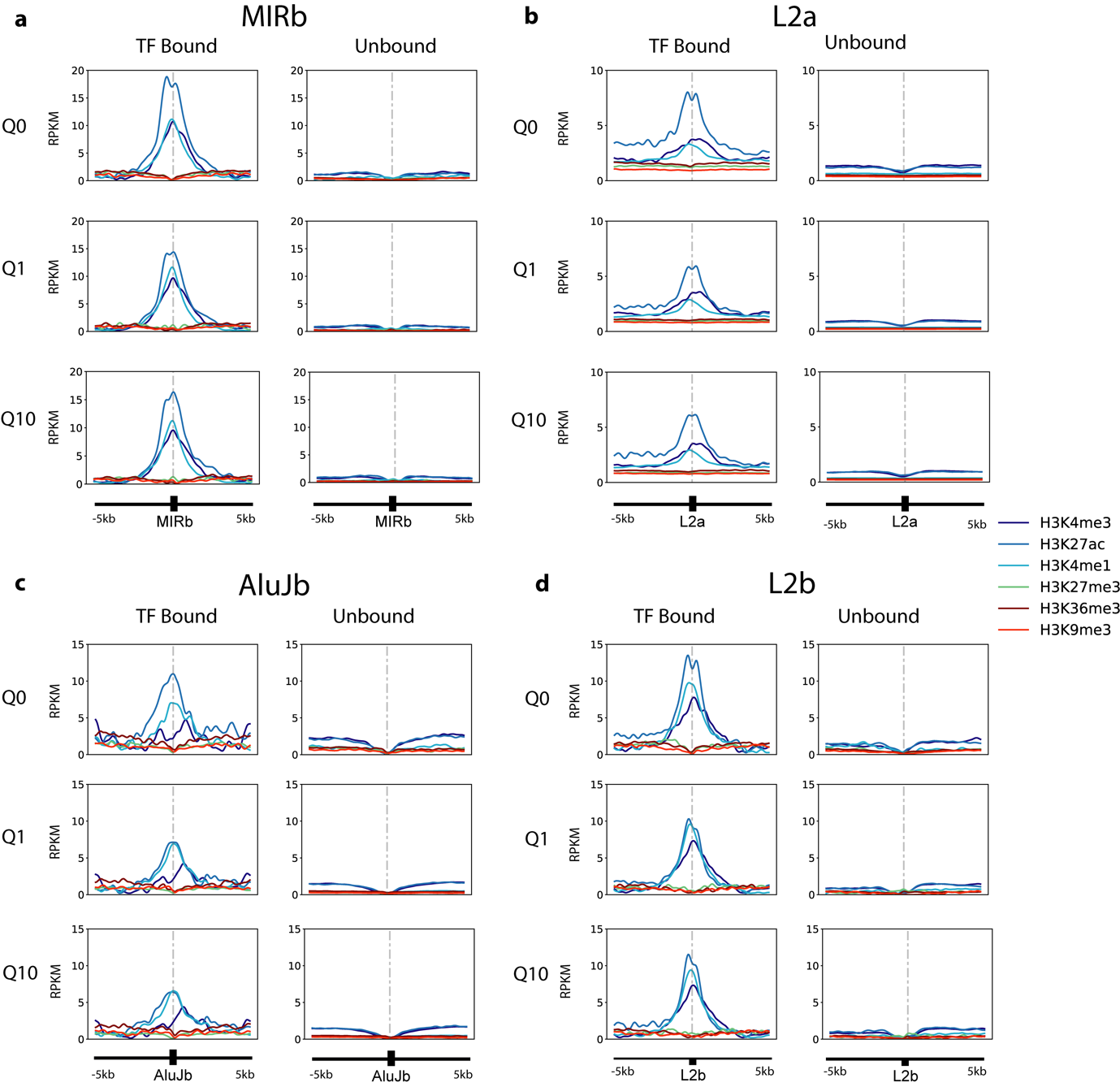
**

**Supplementary Fig.S10.** Comparison of increasing mapQ cutoff on average histone signatures. To ensure that the observed enrichment of active histone profiles around TF bound TE elements was not due to incorrectly placed multi-mapped reads when including all primary alignments (Q0), we repeated enrichment analyses using a mapping quality (mapQ) cut-off of 1 (Q1) or 10 (Q10). These cut-offs excludes reads that map to multiple locations with the same alignment score (Q1), or most reads with multiple possible alignments (Q10). This was performed for repeat subfamilies shown in Fig.1c (**a** and **b**) and Additional File 1: Supplementary Fig.S6 (**c** and **d**). The extent of enrichment is reduced with the mapQ cut-off, however the overall patterns of enrichment are highly conserved with an enrichment of the active histone marks H3K4me3, H3K27ac and H3K4me1 surrounding TF bound TEs, while no enrichment was observed around TEs lacking TF binding. No enrichment was observed for H3K27me3, H3K36me3 or H3K9me3. This result demonstrates that differential histone profiles around TF bound TEs are not an artefact of including multi-mapping reads or downstream processing applied in this study.

**
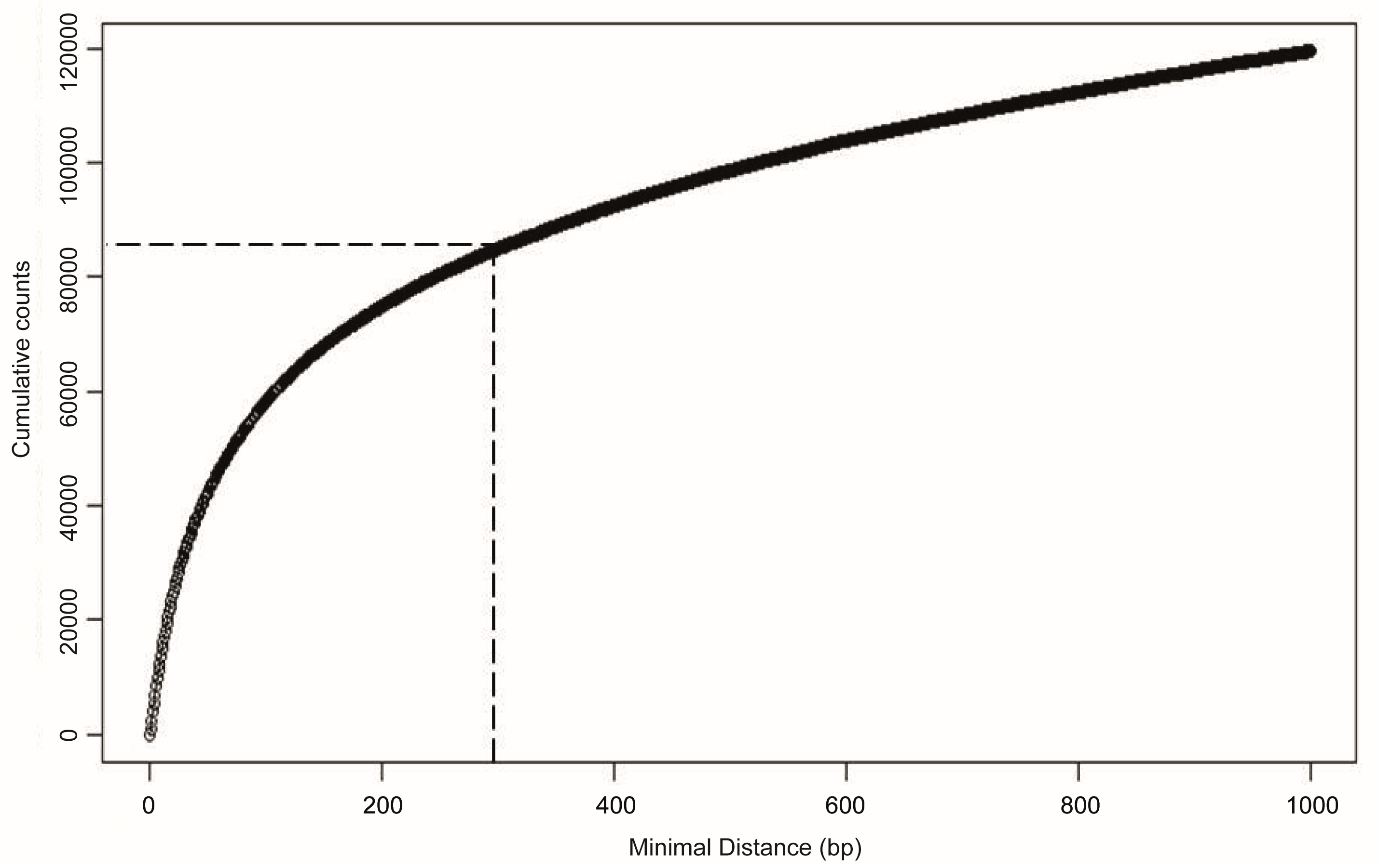
**

**Supplementary Fig.S11.** Cumulative counts of the distances between CAGE clusters and their closest TF binding peaks. For the ChIP-seq datasets analysed in this study, the distance between the middle of each peak and its nearest CAGE cluster was measured. The cumulative counts of distances up to 1kb were plotted. The majority of CAGE clusters were found to be located within 300bp of a TFBS (annotated by the dotted lines).


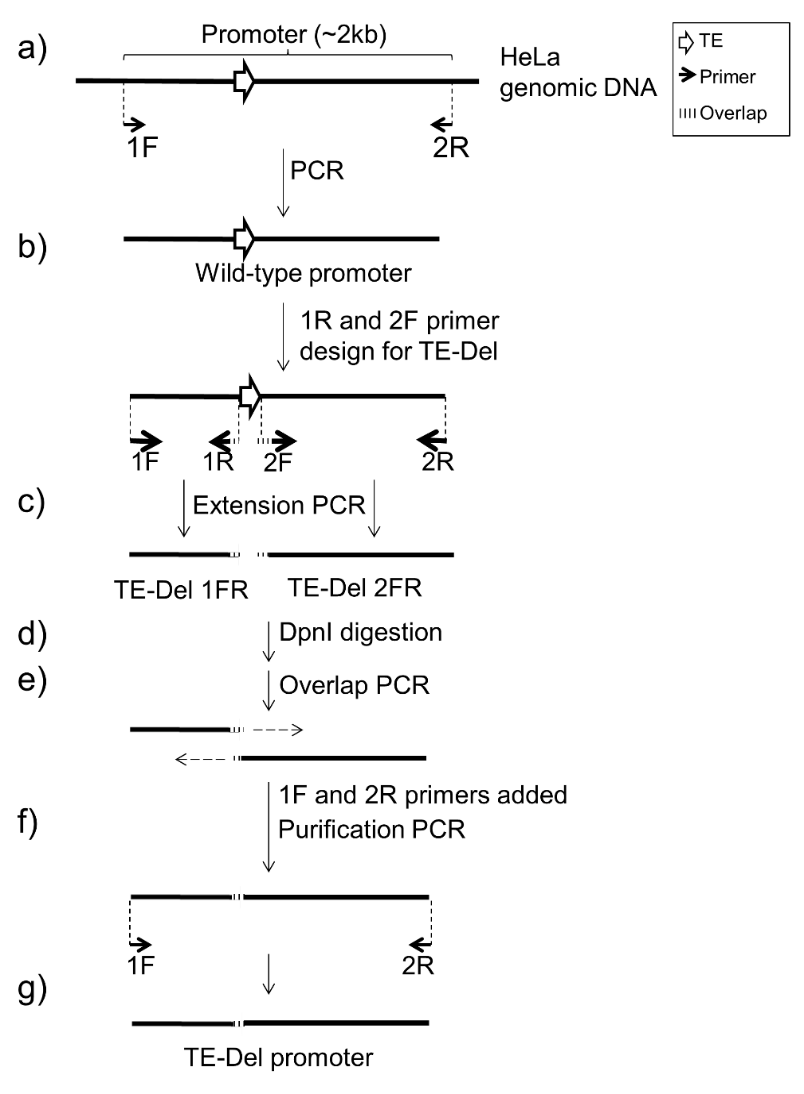


**Supplementary Fig.S12.** Flow chart of molecular cloning of promoters. **a**) For each gene, the wild-type promoter containing the targeted TE was amplified from HeLa genomic DNA by PCR using primers 1F and 2R, and subsequently ligated into the pCR Blunt vector (not shown). PCR-driven overlap extension [5] was conducted to remove the TE from the promoter. **b**) Primers 1R and 2F were designed to flank the deleted region, and to include a complementary overlap region at the 5′ ends (highlighted by dotted lines). **c**) Extension PCR was performed with primers 1F and 1R or primers 2F and 2R to amplify the two intermediate fragments. **d**) Plasmid DNA was removed by DpnI digestion. **e**) In overlap PCR, the overlapped regions were used for the extension of the two fragments. **f**) For purification PCR, primers 1F and 2R were added before further amplification cycles. **g**) The resulting promoter containing the TE deletion (TE-Del) was ligated into the pCR Blunt vectors (not shown).

**Supplementary References**

1. Smit A, Hubley, R & Green, P. RepeatMasker Open-4.0. 2013-2015. <http://www.repeatmasker.org>. Accessed 02 Apr 2017.

2. Severin J, Lizio M, Harshbarger J, Kawaji H, Daub CO, Hayashizaki Y, et al. Interactive visualization and analysis of large-scale sequencing datasets using ZENBU. Nat biotechnol. 2014;32:217-9.

3. Kahles A, Behr J, Rätsch G. MMR: a tool for read multi-mapper resolution. Bioinformatics. 2016;32:770-2.

4. Heinz S, Benner C, Spann N, Bertolino E, Lin YC, Laslo P, et al. Simple combinations of lineage-determining transcription factors prime cis-regulatory elements required for macrophage and B cell identities. Mol Cell. 2010;38:576-89.

5. Heckman KL, Pease LR. Gene splicing and mutagenesis by PCR-driven overlap extension. Nat Protoc. 2007;2:924-32.
