# Supplementary material for: Human transposons are an abundant supply of transcription factor binding sites and promoter activities in breast cancer cell lines
Source: Mob DNA. 2019 Apr 27;10:16. doi: 10.1186/s13100-019-0158-3 (PMC6486989; doi:10.1186/s13100-019-0158-3)
Supplement: Supplementary file 2 — CAGE clusters and genes mapped to putative TE-derived promoters: contains the list of CAGE clusters and genes overlapping TE-derived promoters (DOCX 54 kb) [file 13100_2019_158_MOESM2_ESM.docx]

# Supplementary Tables

**Supplementary Table S1.** Literature evidence on the oncogenicity of candidate genes

| Gene | Product | Literature evidence on functions and associations with cancer |
| --- | --- | --- |
| *SYT1* | Protein | - A Ca^2+^ sensor involved in membrane fusion and neurotransmitter release at synapses [1, 2]. - Modulates export of Fibroblast Growth Factor 1 (FGF-1), a growth factor involved in tumour growth [3]. |
|  |  |  |
| *UCA1* | lncRNA | - Overexpression found in many cancers such as bladder and prostate cancers [4, 5]. - Modulates breast cancer development by exerting an inhibitory effect on tumour suppressors: 1) Inhibits tumour suppressive miR-143 by direct interaction [6]; 2) Inhibits p27, a well-established tumour suppressor, by out-competing it for the binding to heterogeneous nuclear ribonucleoprotein I (hnRNP I), which promotes the translation of the p27 mRNA through binding to its 5′ UTR [7]. |
|  |  |  |
| *AK4* | Protein | - Catalyses phosphoryl transfers among adenine nucleotides and maintains energy homeostasis [8, 9]. - Suggested to modulate drug resistance as knockdown in HeLa cells decreases drug tolerance [8]. - Overexpression correlated with poor prognosis in lung cancer [9]. |
|  |  |  |
|  |  |  |
| *PSAT1* | Protein | - An enzyme involved in serine biosynthesis [10]. - Promotes cell proliferation and tumour formation in *in vitro* studies, and linked to multiple cancers, including colon and non-small cell lung cancer [11]. |
|  |  |  |

**Supplementary Table S2.** Summary of DNase-seq datasets from the ENCODE Consortium [12]. These datasets were generated by the John Stamatoyannopoulos Lab at UW.

| Cell line | Source | ENCODE Accession | GEO Accession |
| --- | --- | --- | --- |
| HMEC | ENCODE | ENCSR000ENV* | GSM736634 |
| MCF7 | ENCODE | ENCSR000EPJ | GSM736588 |

*Only one replicate (ENCFF001DFX) of dataset ENCSR000ENV was analysed as the other one (ENCFF001DFW) exceeded analysis capacity due to its large size.

**Supplementary Table S3.** Summary of ChIP-seq datasets for E2F1, MYC and C/EBPβ conducted on MCF7 cells, acquired from GEO and ENCODE [12, 13]*

| TF | ENCODE Accession | GEO Accession | Control ENCODE Accession | Control GEO Accession |
| --- | --- | --- | --- | --- |
| E2F1 | ENCSR000EWX | GSM935477 | N/A | GSM935485 |
| MYC | N/A | GSM808755/754 | N/A | GSM808758 |
| MYC | ENCSR000DMM | GSM1006877 | ENCSR000DMW | GSM82228 |
| MYC | ENCSR000DMQ | GSM822301 | ENCSR000DMW | GSM82228 |
| MYC | ENCSR000DMJ | GSM1006866 | ENCSR000DMW | GSM82228 |
| C/EBPβ | ENCSR000BSR | GSM1010889 | ENCSR000AHE | GSM1010854 |

*The E2F1 dataset was generated by the Peggy Farnham Lab at USC. The MYC datasets were generated by the Vishwanath Iyer Lab at UTA. The C/EBPβ dataset was generated by the Richard Myers Lab at HAIB.

**Supplementary Table S4.** Sources of histone modification ChIP-seq datasets conducted on MCF7 cells, acquired from the ENCODE Consortium [12]. These datasets were generated by the Bradley Bernstein Lab at the Broad Institute.

| Histone modification | Accession | GEO Accession | Control Accession | GEO Accession |
| --- | --- | --- | --- | --- |
| H3K4me1 | ENCSR493NBY | GSE86714 | ENCSR768LHG | GSE86749 |
| H3K27me3 | ENCSR761DLU | GSE96363 |  |  |
| H3K27ac | ENCSR752UOD | GSE96352 |  |  |
| H3K4me3 | ENCSR985MIB | GSE96506 |  |  |
| H3K36me3 | ENCSR610IYQ | N/A |  |  |
| H3K9me3 | ENCSR999WHE | GSE96517 |  |  |

**Supplementary Table S5.** Sources of mRNA and lncRNA annotations in the human genome, listed in the order of descending priority for annotation of probe targets.

| Genetic element | Database | References |
| --- | --- | --- |
| mRNA | GENCODE (version 24) | Harrow, Frankish [14] |
| lncRNA | The Functional Annotation of the Mammalian Genome (FANTOM) CAGE associated transcriptome (FANTOM CAT) | Hon, Ramilowski [15] |
| lncRNA | NONCODE | Zhao, Li [16] |

**Supplementary Table S6.**  Primer sequences used in PCR reactions and sequencing. The restriction enzyme (RE) recognition sites are underlined.

| Primer name | RE | Sequence (5′ to 3′) |
| --- | --- | --- |
| SYT1_1F | MluI | TATTAACGCGTAACCTTTCTCTCTGGCTGC |
| SYT1_2R | MluI | TATTAACGCGTGGTTTGCCTGACTGC |
| SYT1_Nested_1F | MluI | TATTAACGCGTCTTTGTGGCATTCTCTGTATTTCC |
| SYT1_Nested_2R | MluI | TATTAACGCGTGGTAAACAGTGACACAGAGC |
| SYT1_L1PA2_deletion_1R | N/A | GTGGTAGAATCAGAGCCCACCACAGC |
| SYT1_L1PA2 _deletion_2F | N/A | TGTGGTGGGCTCTGATTCTACCACCAAGAATAAAATAGTTG |
| SYT1_Mid_Seq | N/A | GTGTGAGGTGTCAGTGTGC |
| UCA1_1F | MluI | TATTAACGCGTGAGTTTGAGGTCAGCC |
| UCA1_2R | BglII | TATTAAGATCTTGGGATGAGTCACATTGGGAGC |
| UCA1_LTR7C__deletion_1R | N/A | TTTGGCACCAAGGTTCCCTTTTGGAAACGTTACAG |
| UCA1_LTR7C_ deletion_2F | N/A | CCAAAAGGGAACCTTGGTGCCAAAATCTGG |
| UCA1_Mid_Seq | N/A | CCCGATTTGAAATTGGTGAGATGTTCC |
| AK4_1F | KpnI | TATTAGGTACCCACCAACCCCAATCAAAGAGC |
| AK4_2R | HindIII | TATTAAAGCTTCCTACCTCTGGCACCCTCC |
| AK4_MIRb _deletion_1R | N/A | GCAAGGGAAGGGCATTGTGTTTACAGCCGAGGAATGG |
| AK4_MIRb _deletion_2F | N/A | CTGTAAACACAATGCCCTTCCCTTGCAGTAGACC |
| AK4_Mid_Seq | N/A | AATAGTTTCTGGCACACAGTAGGC |
| PSAT1_1F | KpnI | TATTAGGTACCGTGTGACTTGTGTCCCTGAAATACC |
| PSAT1_2R | XhoI | TATTACTCGAGGAGTCAGCCAAGGAGGACC |
| PSAT1_MIR3_ deletion_1R | N/A | ATGCCCGCCAGAAACGTGAGGAGATATTAACAAGAACG |
| PSAT1_MIR3_ deletion_2F | N/A | CTCACGTTTCTGGCGGGCATCCACG |
| PSAT1_Mid_Seq | N/A | CCAGACTTCAAAGGAACTTCTTTCTTGC |

**Supplementary Table S7.** Plating density for the TNBC cell lines on a 24-well plate.

| TNBC cell line | Plating density (cells per well)* |
| --- | --- |
| MDA-MB-468 | 0.45 x 10^6^ |
| MDA-MB-231 | 0.3 x 10^6^ |
| BT549 | 0.1 x 10^6^ |

***** These plating densities led to an 80%-90% confluency at the time of transfection.

**Supplementary References**

1. Lai Y, Lou X, Diao J, Shin Y-K. Molecular origins of synaptotagmin 1 activities on vesicle docking and fusion pore opening. Sci Rep. 2015;5:9267.

2. Sudhof TC. A molecular machine for neurotransmitter release: synaptotagmin and beyond. Nat Med. 2013;19:1227-31.

3. Egan JB, Barrett MT, Champion MD, Middha S, Lenkiewicz E, Evers L, et al. Whole genome analyses of a well-differentiated liposarcoma reveals novel SYT1 and DDR2 rearrangements. PLoS ONE. 2014;9:e87113.

4. Zhang S, Dong X, Ji T, Chen G, Shan L. Long non-coding RNA UCA1 promotes cell progression by acting as a competing endogenous RNA of ATF2 in prostate cancer. Am J Transl Res. 2017;9:366-75.

5. Zhen S, Hua L, Liu YH, Sun XM, Jiang MM, Chen W, et al. Inhibition of long non-coding RNA UCA1 by CRISPR/Cas9 attenuated malignant phenotypes of bladder cancer. Oncotarget. 2017;8:9634-46.

6. Tuo YL, Li XM, Luo J. Long noncoding RNA UCA1 modulates breast cancer cell growth and apoptosis through decreasing tumor suppressive miR-143. Eur Rev Med Pharmacol Sci. 2015;19:3403-11.

7. Huang J, Zhou N, Watabe K, Lu Z, Wu F, Xu M, et al. Long non-coding RNA UCA1 promotes breast tumor growth by suppression of p27 (Kip1). Cell Death Dis. 2014;5:e1008.

8. Fujisawa K, Terai S, Takami T, Yamamoto N, Yamasaki T, Matsumoto T, et al. Modulation of anti-cancer drug sensitivity through the regulation of mitochondrial activity by adenylate kinase 4. J Exp Clin Cancer Res. 2016;35:48.

9. Jan YH, Tsai HY, Yang CJ, Huang MS, Yang YF, Lai TC, et al. Adenylate kinase-4 is a marker of poor clinical outcomes that promotes metastasis of lung cancer by downregulating the transcription factor ATF3. Cancer Res. 2012;72:5119-29.

10. Liao KM, Chao TB, Tian YF, Lin CY, Lee SW, Chuang HY, et al. Overexpression of the PSAT1 gene in nasopharyngeal carcinoma Is an indicator of poor prognosis. J Cancer. 2016;7:1088-94.

11. Liu B, Jia Y, Cao Y, Wu S, Jiang H, Sun X, et al. Overexpression of phosphoserine aminotransferase 1 (PSAT1) predicts poor prognosis and associates with tumor progression in human esophageal squamous cell carcinoma. Cell Physiol Biochem. 2016;39:395-406.

12. ENCODE Project Consortium. An integrated encyclopedia of DNA elements in the human genome. Nature. 2012;489:57-74.

13. Barrett T, Wilhite SE, Ledoux P, Evangelista C, Kim IF, Tomashevsky M, et al. NCBI GEO: archive for functional genomics data sets—update. Nucleic Acids Res. 2013;41:D991-5.

14. Harrow J, Frankish A, Gonzalez JM, Tapanari E, Diekhans M, Kokocinski F, et al. GENCODE: The reference human genome annotation for The ENCODE Project. Genome Res. 2012;22:1760-74.

15. Hon C-C, Ramilowski JA, Harshbarger J, Bertin N, Rackham OJL, Gough J, et al. An atlas of human long non-coding RNAs with accurate 5′ ends. Nature. 2017;543:199-204.

16. Zhao Y, Li H, Fang S, Kang Y, Wu W, Hao Y, et al. NONCODE 2016: an informative and valuable data source of long non-coding RNAs. Nucleic Acids Res. 2016;44:D203-8.
